# Alpha-Glucan, Water Dikinase 1 Affects Starch Metabolism and Storage Root Growth in Cassava (*Manihot esculenta* Crantz)

**DOI:** 10.1038/s41598-017-10594-6

**Published:** 2017-08-29

**Authors:** Wenzhi Zhou, Shutao He, Maliwan Naconsie, Qiuxiang Ma, Samuel C. Zeeman, Wilhelm Gruissem, Peng Zhang

**Affiliations:** 1 National Key Laboratory of Plant Molecular Genetics, CAS Center for Excellence in Molecular Plant Sciences, Institute of Plant Physiology and Ecology, Shanghai Institutes for Biological Sciences, Chinese Academy of Sciences, 200032 Shanghai, China; 2 Institute of Agricultural Sciences, Department of Biology, ETH Zurich, 8092 Zurich, Switzerland

## Abstract

Regulation of storage root development by source strength remains largely unknown. The cassava *storage root delay* (*srd*) T-DNA mutant postpones storage root development but manifests normal foliage growth as wild-type plants. The *SRD* gene was identified as an orthologue of α-glucan, water dikinase 1 (GWD1), whose expression is regulated under conditions of light/dark cycles in leaves and is associated with storage root development. The GWD1-RNAi cassava plants showed both retarded plant and storage root growth, as a result of starch excess phenotypes with reduced photosynthetic capacity and decreased levels of soluble saccharides in their leaves. These leaves contained starch granules having greatly increased amylose content and type C semi-crystalline structures with increased short chains that suggested storage starch. In storage roots of GWD1-RNAi lines, maltose content was dramatically decreased and starches with much lower phosphorylation levels showed a drastically reduced β-amylolytic rate. These results suggested that GWD1 regulates transient starch morphogenesis and storage root growth by decreasing photo-assimilation partitioning from the source to the sink and by starch mobilization in root crops.

## Introduction

Cassava (*Manihot esculenta* Crantz) is an important root crop that accumulates large amounts of starch in its storage roots, which is extensively cultivated in the tropics and subtropics^1, 2^. Cassava is also recognized for its drought and infertility tolerance in marginal lands^3, 4^. During cassava growth, photosynthetically-derived carbohydrates are appropriately partitioned from the leaves to storage roots, which are the major sink organs that reflects both cassava yield and starch productivity^5, 6^. Unlike grain crops, photo-assimilates in cassava are translocated from the leaves to storage roots – an important process that contributes to sink productivity, although the regulatory mechanism is largely unknown^7, 8^.

The supply of carbon from transient starch through photosynthesis is vital for plant growth. At night, transient starch is degraded to provide substrates for respiration and sucrose synthesis^9^. Although carbon partitioning between daytime transient starch biosynthesis and night-time starch degradation has been extensively studied in Arabidopsis, several enzymes associated with transient starch degradation, including amylase, 4-alpha-glucanotransferase and isoamylase, are non-essential for flux control^10, 11^. Instead, starch phosphorylation and dephosphorylation play an essential role in starch turnover in higher plants^12, 13^. Mutants that are defective in starch phosphorylation and dephosphorylation inhibit transient starch degradation in leaves, which demonstrates a starch excess phenotype with compromised growth^14^. However, seed-specific reductions in starch phosphorylation increases the yield in maize and barley, which suggests that starch phosphorylation plays a role in carbon partitioning between the source and the sink^15, 16^.

Generally, the presence of monoesterified phosphate groups at the C6 and C3 positions of the glucosyl residue is catalyzed by glucan water dikinase (GWD) and phosphoglucan water dikinase (PWD), respectively. This biochemical reaction is completed by transferring the β-phosphate of ATP to the substrate and the γ-phosphate to water^17^. Phosphorylation by GWD and PWD unwinds the double helical structure, facilitating the ability of β-amylase and isoamylase to degrade surface glucans. The phosphate groups are subsequently removed by phosphatase like SEX4 and LSF2 as β-amylase cannot bypass the phosphate groups^13^. GWD was first studied in the potato as the chloroplastic starch-related R1 protein that is responsible for starch phosphorylation, which can also repress cold sweetening^17–19^. Its reduced expression results in a starch excess phenotype in mature leaves, leading to an increase in dry matter^18^. Overexpression of potato GWD in barley increased starch-bound phosphate content in barley caryopsis starch^20^. Starch phosphorylation also affects size, morphology and thermal properties of starch granules by affecting the activities of other starch metabolic enzymes in plants^21–23^.

In Arabidopsis, mutant plants that lacked GWD or PWD accumulated significant levels of excess starch and showed retarded growth. However, a recent study claimed that these enzymes exhibited a very low flux control coefficient with respect to starch synthesis and degradation in the leaves^11^. Constitutive suppression of GWD expression in maize and rice showed no impact on vegetative biomass^16, 24^. Increased vegetative biomass and yield were reported in wheat following dampened activity of GWD in its endosperm^15, 25^. In addition, no severe phenotypic changes and no effect on starch accumulation in the tubers were observed in GWD antisense potato lines^18^. Moreover, similar to the potato, cassava has an above-ground leaf source and an under-ground storage root sink, which results in long-distance downward carbon transport and partitioning between the leaves and storage roots^5, 26^. Investigating its source strength, especially transient starch turnover, increases our understanding of the mechanisms that underlie the source-sink relationships in root crops. Further, the role of starch phosphorylation in starch metabolism needs to be determined in transient and storage starches of the cassava.

In the current study, we have identified the *storage root delay* (*srd*) cassava mutant, which showed retarded storage root growth both in the greenhouse and the field. The mutation was traced to a T-DNA insertion in the eighth exon of α-glucan, water dikinase 1 gene (*MeGWD1*) in the cassava genome, which leads to reduced MeGWD1 production. Down-regulation of *MeGWD1* expression in transgenic cassava by RNAi confirmed the starch excess phenotype in the leaves and retarded storage root growth. Consequently, physico-chemical properties of transient and storage starches and sugar content in GWD1-RNAi leaves and storage roots are also affected. We showed that the cassava GWD1 gene regulates transient starch morphogenesis and degradation in source and carbon partitioning by modulating starch phosphorylation.

## Results

### Cassava *srd* mutant is a T-DNA insertion line of the α-glucan, water dikinase gene

**Figure 1:**
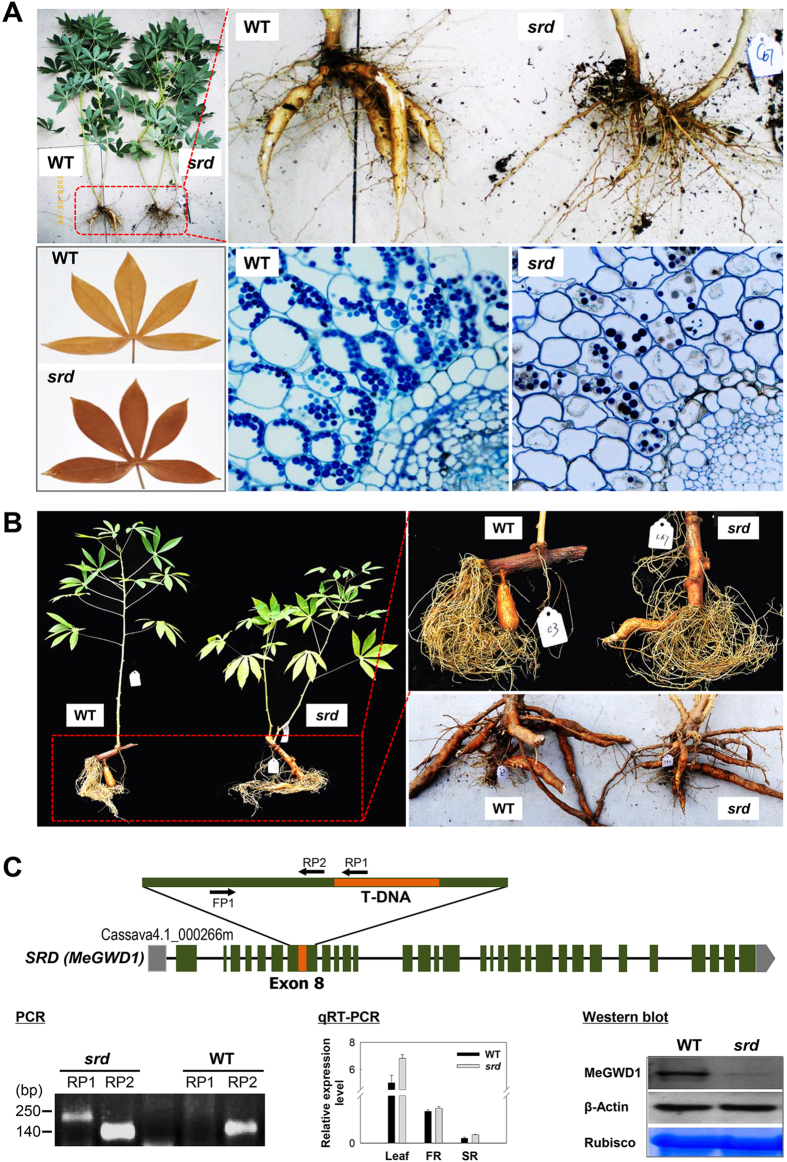
Phenotypic and molecular characterization of cassava *srd* mutant. (**A**) Normal foliage development of wild type (WT) and *srd* mutants and delay of storage root formation in *srd* mutants after three months of growth in the greenhouse using shoot culture; no obvious change in mature leaves at the end of the dark period after iodine staining were noted; by contrast, the young stem of the *srd* mutant showed markedly decreased starch accumulation in cortical parenchyma cells than in the WT. (**B**) Reduced growth of storage roots in the *srd* mutant in the greenhouse (left and upper-right panels) and field-grown (bottom right panel) plants using stem cuttings. (**C**) Verification of the T-DNA insert by PCR using the primer pair FP1/RP1 and FP1/RP2, and qRT-PCR using the primer pair G1qrt5’F/G1qrt5′R and following Western blot using a specific antibody directed against MeGWD1, where it was found that the T-DNA insert in the eighth exon of MeGWD1 led to reduced protein expression.

The cassava *storage root delay* (*srd*) mutant was identified after screening a cassava T-DNA population of more than 300 lines that were grown under the conditions of greenhouse. After three months of growth using *in vitro* shoot cultures as planting material, the *srd* plants showed normal foliage development but lacked storage roots (Fig. 1A, upper panels). Iodine staining assays of the leaves at the end of the light period showed no obvious phenotypic difference in starch accumulation between the wild type (WT) and the *srd* mutant (Fig. 1A, bottom left panel), except more reddish brown in the mutant. Furthermore, transverse sectioning of very young stems with iodine staining showed that the cortical parenchyma contained an abundance of starch granules in the WT as compared the *srd* mutant (Fig. 1A, bottom right panel) - a similar observation was found in free hand-cut sections of their young stems (Supplementary Fig. 1). After planting mature stem cuttings from field harvested plants in pots for three months, it was found that the *srd* mutant produced smaller storage roots than did the WT (Fig. 1B, left and upper right panels; Supplementary Fig. 2). A reduced fresh weight (approximately 50% of WT, 1.7 kg/plant) of storage roots in the field was also observed when harvested (Fig. 1B, bottom right panel). After confirming single T-DNA integration by Southern blot analysis (data not shown), the T-DNA flanking sequences of the *srd* mutant were amplified by inverse-nested PCR and sequenced. Alignment of the flanking sequences with the cassava genome indicated that the T-DNA was inserted into the eighth exon of the α-glucan, water dikinase 1 gene (*MeGWD1*) in one homologous chromosome, which was confirmed by PCR analysis using two sets of primer pairs (Fig. 1C, bottom left panel; Supplementary Table 1). Although the *MeGWD1* transcript level was unaffected in the leaves or in the fibrous and storage roots by quantitative reverse transcription polymerase chain reaction (qRT-PCR) analysis (Fig. 1C, bottom middle panel), its protein level was decreased as shown by Western blotting analysis using an anit-MeGWD1 specific antibody (Fig. 1C, bottom right panel; Supplementary Fig. 3). An approximate 65% decrease in MeGWD1 protein was detected in the heterologous mutant.

### Cassava has three GWD homologous genes belonging to two different groups

**Figure 2:**
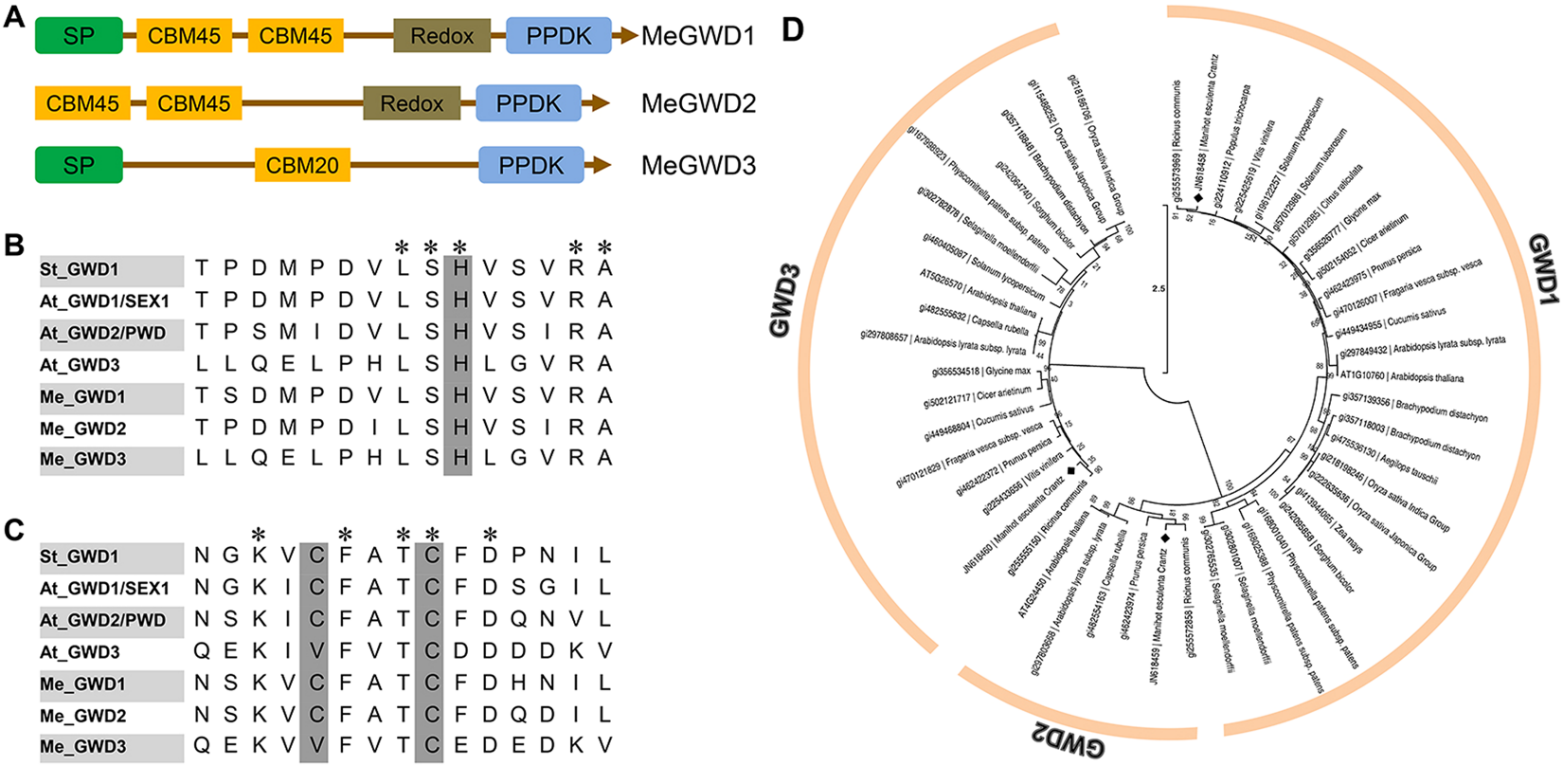
Sequence analysis of cassava GWDs. (**A**) Alignment of regions surrounding the putative phosphohistidines of GWDs from cassava, potato and Arabidopsis. (**B**) Alignment of regions surrounding the regulatory CFATC sequence of cassava, potato and Arabidopsis. (**C**) Putative conserved domains of cassava GWDs. (**D**) Phylogenetic analysis of GWDs from various species using neighbor-joining method by MEGA5. Cassava GWDs are marked by the black rhombus.

Three cassava GWD genes, which are referred to as *MeGWD1*, *MeGWD2* and *MeGWD3*, were identified after blast searching the cassava genome (http://www.phytozome.net) and the cDNA database of RIKEN^27^ (http://en.brc.riken.jp) using potato and Arabidopsis GWD1 protein sequences as queries. Their full open-reading frames (ORFs) were 4230 bp, 3687 bp and 3585 bp in length, respectively, and were cloned from cassava cDNAs. The MeGWD1, MeGWD2 and MeGWD3 proteins are made up of 1410, 1229, and 1195 amino acids, respectively (Supplementary Table 2). The MeGWD1 and MeGWD3 proteins were predicted to be chloroplastic proteins with a signal peptide at the N-terminus, which was similar to that shown for GWD1 and GWD3 from Arabidopsis; however, MeGWD2 lacked a signal sequence as analyzed by ChloroP^28^ (Fig. 2A). The C-terminus of the cassava GWDs displayed homology to the nucleotide binding domain of pyruvate, phosphate dikinase^29^ (PPDK, EC 2.7.9.1, Fig. 2B). Two tandem carbohydrate binding modules 45 (CBM45) were also located at the N-terminus of MeGWD1 and MeGWD2 according to the CAZY database (http://www.cazy.org/). Further, MeGWD3 was predicted to have a single carbohydrate binding module 20 (CBM20), which was similar to the GWD3 of Arabidopsis and potato^30, 31^ (Fig. 2A). The redox-regulation motif (CFATC) was also found in MeGWD1 and MeGWD2, as was the potato GWD sequence, which suggested that MeGWD1 and MeGWD2 were also redox-regulated^32^. The first cysteine in the redox motif was replaced by valine in MeGWD3, which was similar to that reported for AtGWD3^32^ (Fig. 2C).

Phylogenetic analysis of the deduced protein sequences of cassava GWDs with other plant GWDs showed that the GWD protein family comprises two groups referred to as GWD1 and GWD3 (Fig. 2D). Group 1 contains two subgroups. The members of the GWD1 subgroup consisted of 25 proteins including those derived from Arabidopsis, potato and cassava. When compared with AtGWD1, MeGWD1 was closer to StGWD1. Furthermore, members of the GWD2 sub-group contained fewer reported members with only six proteins (including MeGWD2) investigated in this study. Group 2 contained 18 proteins of GWD3. Interestingly, GWD1 and GWD3 were identified in most species, with GWD2 absent in some of them.

### Transcription of cassava GWD genes is associated with diurnal oscillation of starch metabolism in leaves and in storage root growth

**Figure 3:**
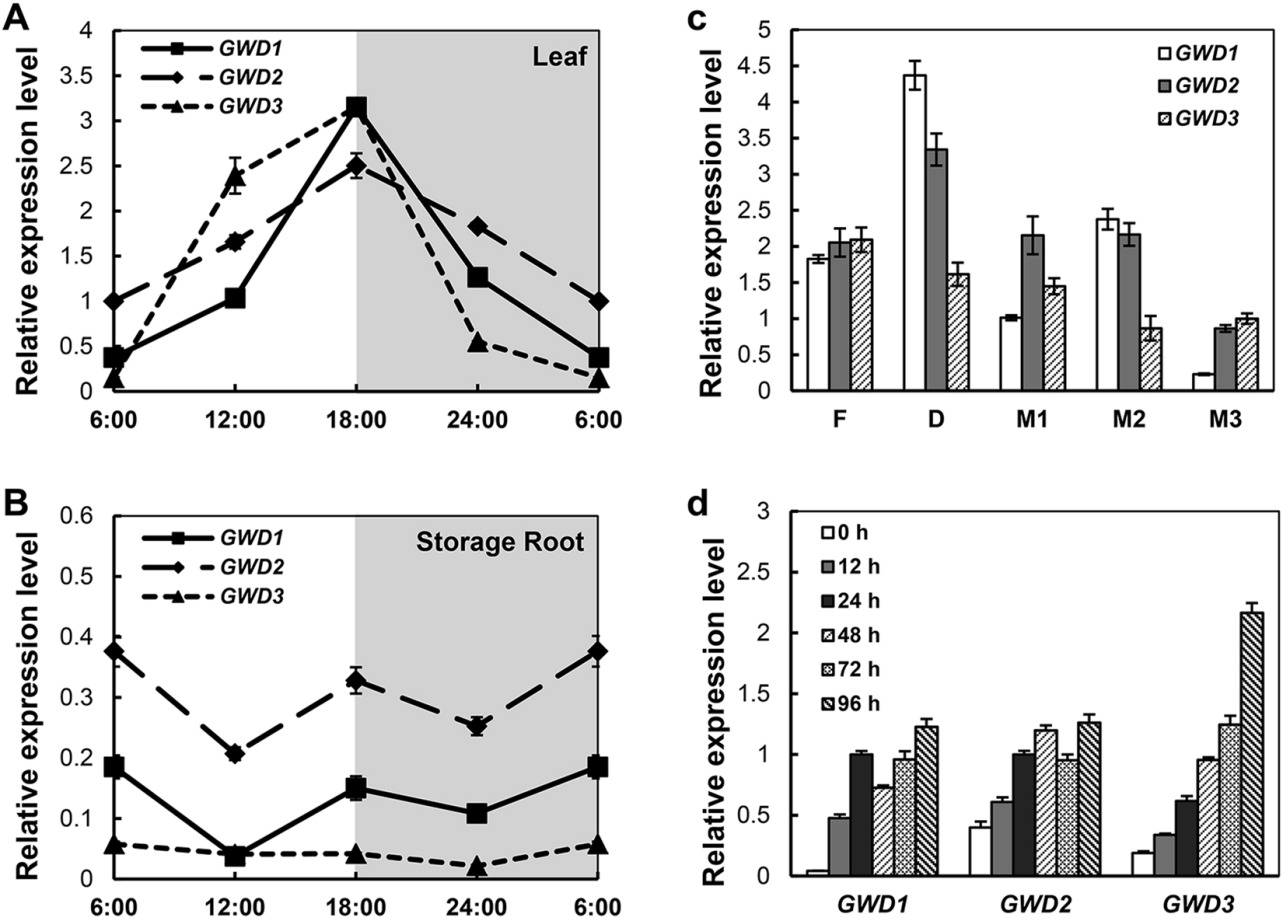
Expression profiles of *GWD*s in cassava. (**A,B**) Responses to light/dark cycles in the mature leaf and storage root of the cassava. (**C**) Fibrous root (F), developing storage root (D), and mature storage roots of early stage (M1), medium stage (M2) and later stage (M3) of development. (**D**) Increased expression of cassava GWDs in storage roots after harvest. The cassava β-actin gene was used as an internal control. Data are presented as mean ± SD of three biological replicates.

The expression pattern of cassava GWD genes responding to different developmental stages and environmental influences was analyzed in cassava using specific primer pairs (Supplementary Table 1 and Supplementary Fig. 4). The GWD transcripts were constantly increased throughout the photoperiod to a maximum at the end of the light cycle, which then decreased during the following dark period of the circadian rhythm (Fig. 3A) - an observation that was consistent with transient starch metabolic rhythm in the leaves^12, 33^. Their mRNA levels were approximately three-fold higher than that of the β-actin gene in cassava leaves. At the end of the dark cycle, the expression of cassava *GWDs* showed the lowest levels of expression. A subset of the *MeGWD1* promoter elements identified with PLACE indicated that GWD1 expression was also regulated by hormones and environmental factors (Supplementary Fig. 5). Unlike those observations found in the leaves, the expression of cassava GWDs in the roots did not respond to the circadian rhythm and their mRNA levels were also low when compared to that of leaves. In addition, the lowest *GWD* expression levels were found for *MeGWD3* (Fig. 3B).

To further check the expression levels of cassava GWDs related to storage root development, roots from five developmental stages (Supplementary Table 3) were analyzed. The *MeGWD1* and *MeGWD2* genes showed increased expression in developing storage roots. In late mature storage roots (M3), cassava GWDs showed the lowest levels of expression as compared roots from other stages. A slight increase in *MeGWD1* transcription was observed in M2 storage roots as compared that of M1 and M3 storage roots (Fig. 3C). This indicates that GWDs are involved in storage root development and starch accumulation. Further, cassava storage roots undergo rapid post-harvest physiological deterioration (PPD) after harvest. During PPD, all three cassava GWDs showed increased expression, which indicated that GWDs are possibly involved in storage starch degradation during PPD (Fig. 3D).

### MeGWD1 expression is related to starch phosphorylation

**Figure 4:**
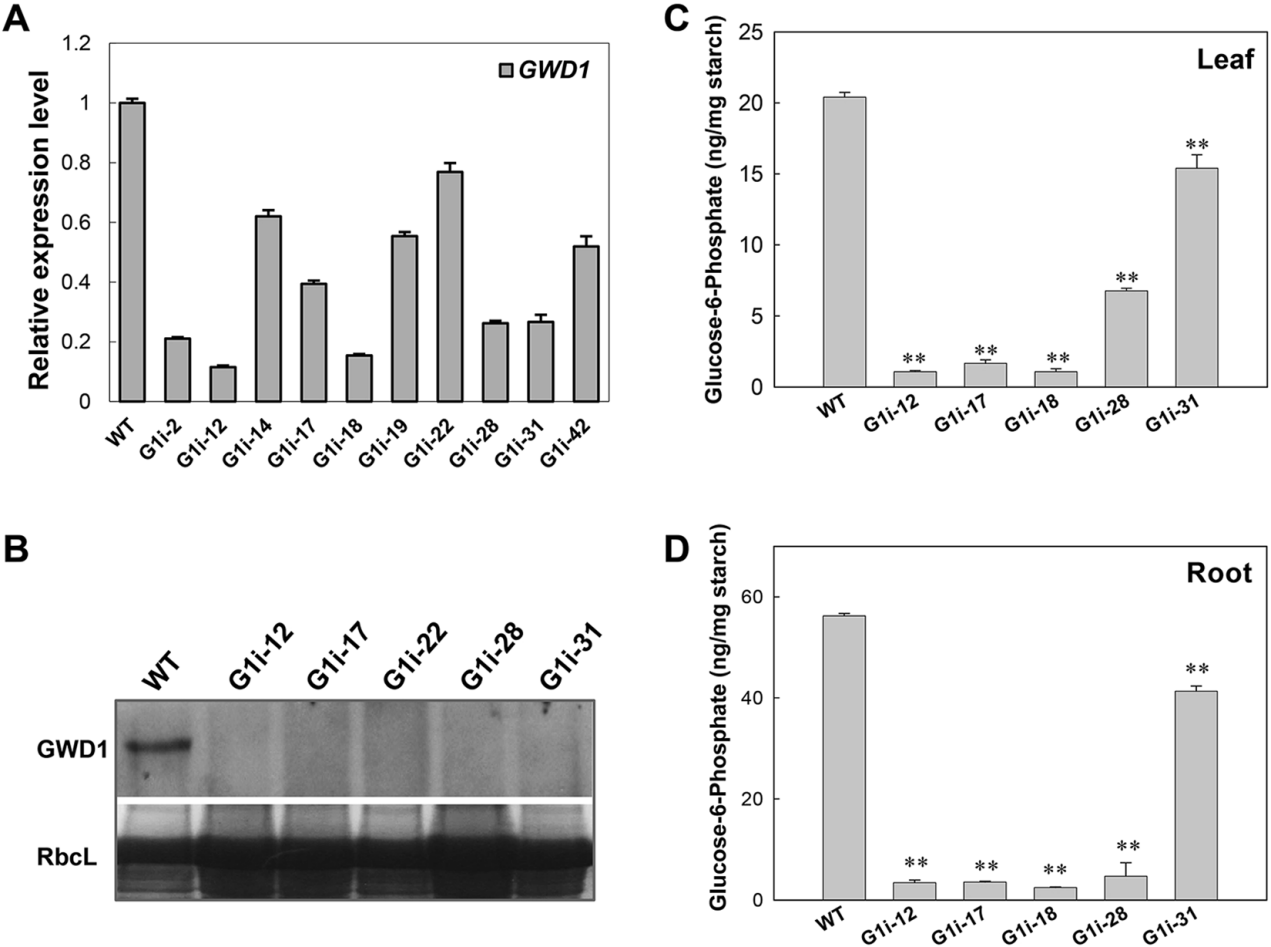
MeGWD1 expression and starch phosphorylation analysis in wild-type (WT) and G1i transgenic cassava plants. (**A**) Relative mRNA levels of GWD1 in mature leaves as determined by qRT-PCR. Cassava β-actin gene expression was used as an internal control. (**B**) Immuno-detection of MeGWD1 in mature leaves of WT and selected G1i transgenic plants with an anti-MeGWD1 antibody. (**C,D**) HPAEC-PAD analysis of starch phosphorylation levels in mature leaves (**C**) and roots (**D**) of WT and G1i transgenic lines, after acid treatment. Values are presented as mean ± SD of three biological replicates. Significant differences were determined by the Student’s *t*-test (**P < 0.05).

To further elucidate the role of GWD1 on storage root development, transgenic cassava was produced with the aim of repressing *MeGWD1* expression by transformation with the hair-pin dsRNA expression cassette 35S::GWD1-RNAi. More than a dozen transgenic cassava lines (named G1i for short) were regenerated. The integration pattern of transgenes in these lines was assessed by Southern blot analysis using the digoxigenin-labelled hygromycin phosphor-transferase gene (*HPT*) probe after genomic DNA *Hin*dIII-digestion (Supplementary Fig. 6). The expression of *MeGWD1* in selected G1i plant lines that had a single T-DNA integration was assessed by qRT-PCR assay. The *MeGWD1* mRNA was decreased by 10–70% in these G1i lines as compared with that of the WT (Fig. 4A). By contrast, the expression of cassava GWD2 and GWD3 was significantly unaffected (Supplementary Fig. 7). Five G1i lines with reduced *MeGWD1* transcription were selected for subsequent analyses. When the protein levels in the five G1i lines were monitored with antisera against cassava MeGWD1 by Western blot analysis, an approximate ~150KDa band was detected in the WT, but not in transgenic lines (Fig. 4B). The size of the band was consistent with the size calculated from the deduced amino acid sequence, which was 149.4 kDa for the mature protein after signal peptide (1–77 aa) cleavage. The results indicated that *MeGWD1* expression in the G1i lines was dramatically decreased, and showed higher levels of stronger repression than the *srd* cassava mutant. These G1i plants showed weak growth from stem cuttings and retarded storage root growth in pots when compared with the WT and the *srd* mutant when grown under greenhouse conditions (Supplementary Fig. 2).

Phosphate levels of transient starches from leaves and storage starches from storage roots were analyzed by HPAEC-PAD after acid hydrolysis. In the WT, phosphorylation level of root storage starch was three times higher than that found in transient starch of leaves (Fig. 4C,D). The Glc-6-P content in WT leaf transient starch and WT storage root starch was 20 ng/mg and 56 ng/mg, respectively. Among the five Gli lines, significant reduction of Glc-6-P was detected in all lines of both the leaf transient starch and storage root starch, with a few lines showing reduced detection limits; e.g., as found for G1i-12, G1i-17 and G1i-18 (Fig. 4C,D). The sharp reduction in starch phosphorylation at the C6 position in the G1i transgenic cassava confirms that *MeGWD1* controls starch phosphorylation in leaves and roots. The higher G1i-28 and G1i-31 transcription levels than was found in other G1i lines revealed relatively lower reductions in starch phosphorylation.

### MeGWD1 is essential for transient starch turnover

**Figure 5:**
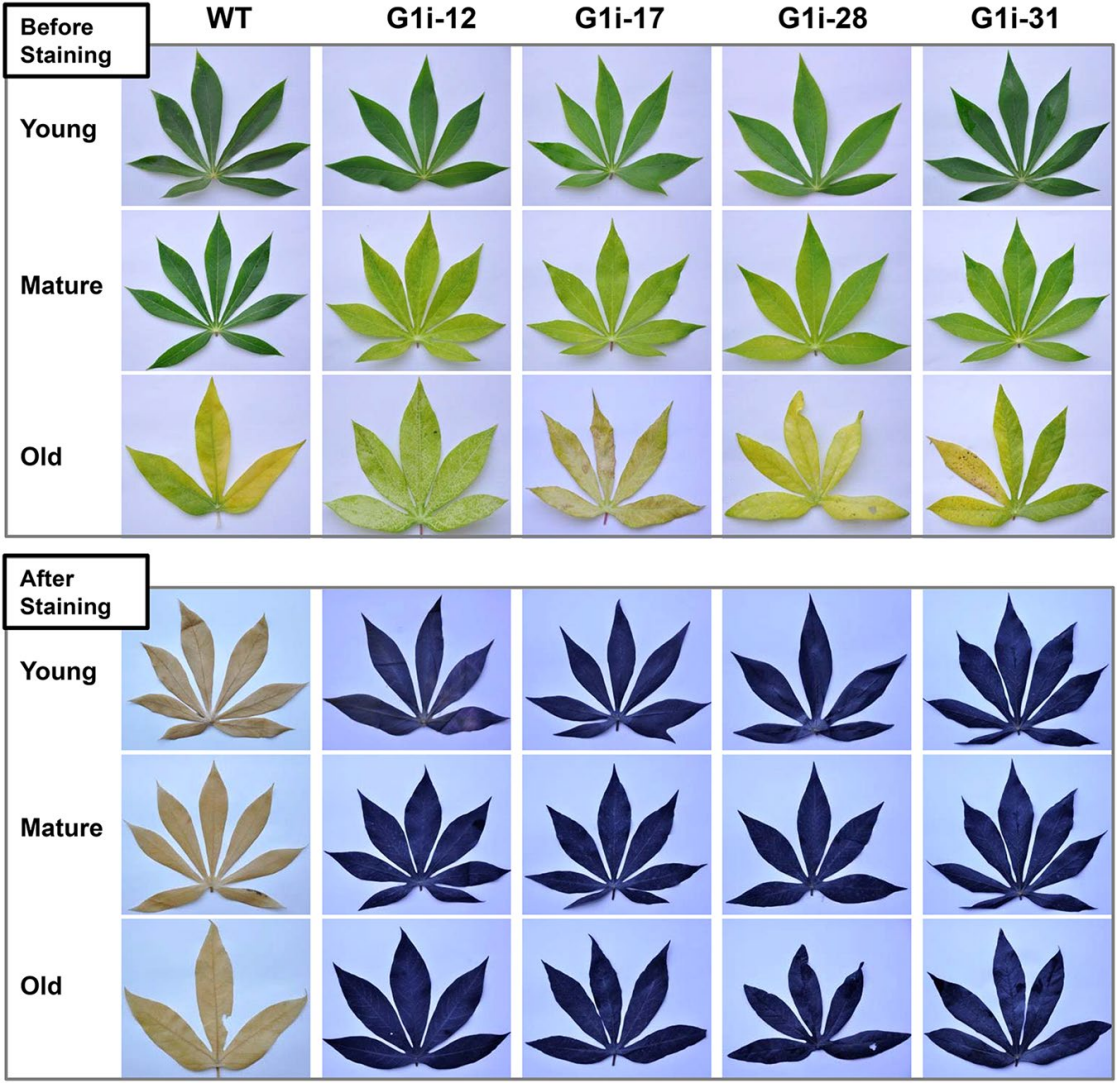
Starch excess phenotype in young, mature and old leaves of G1i transgenic cassava vs. the wild type (WT), at the end of the dark period after iodine staining.

The starch excess phenotype in leaves that was found at different developmental stages was investigated at the end of the light period. Compared with WT leaves, mature leaves of G1i lines were yellowish, especially in the G1i-12, -17 and -28 lines. The G1i-31 line displayed a moderate change of leaf senescence (Fig. 5, upper panel). After staining with iodine solution, all leaves of G1i lines were dark blue, which implied a typical starch-excess phenotype. By contrast, no such observation was seen in young, mature and old WT leaves (Fig. 5, lower panel).

**Figure 6:**
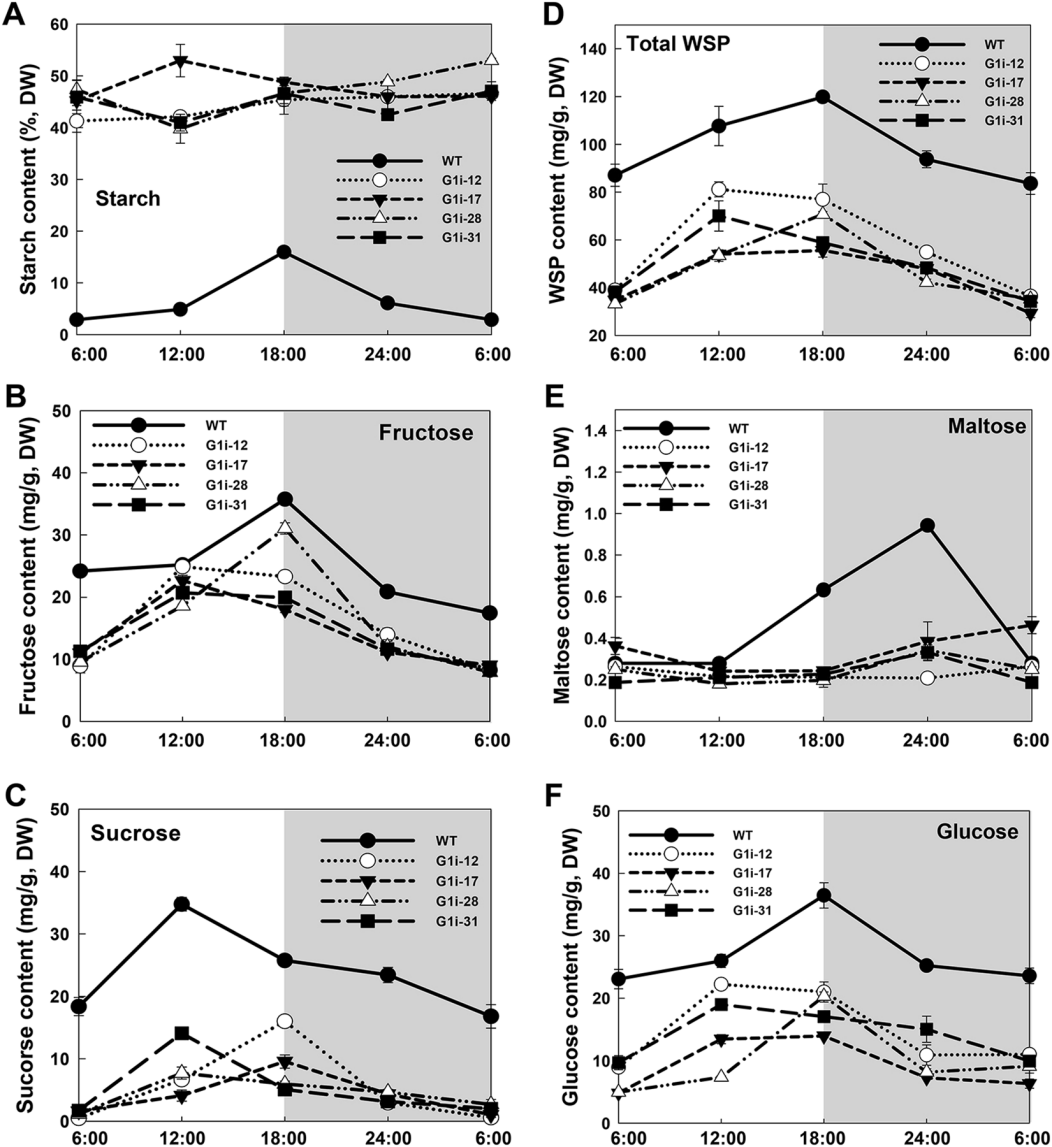
Diurnal changes of sugar and starch content in wild-type (WT) and G1i transgenic cassava. (**A**) Glucose; (**B**) Fructose; (**C**) Sucrose; (**D**) Water soluble polysaccharides (WSP); (**E**) Maltose; (**F**) Starch. Values are presented as mean ± SD of three biological replicates.

Furthermore, when compared with WT that increased in light and decreased in dark cycles, the G1i transgenic lines maintained a constantly high level of starch content in their leaves. The starch content in transgenic lines reached more than 40% DW, which was ten-fold higher than was found in WT leaves at the beginning of the light period. At the end of the day, the differences in leaf starch content between WT and G1i transgenic lines remained at approximately three-fold difference (Fig. 6A).

Significant changes were also detected in soluble sugars including glucose, fructose, sucrose, maltose and water soluble polysaccharides (WSP) in the leaves of G1i lines (Fig. 6B–F). A significant reduction in sugar level was found in the leaves of G1i transgenic plants during the day/night cycle. Overall, all sugars, with the exception of maltose, were reduced to a minimum of half the level found in WT plants in the G1i leaves at the end of the dark phase. At the conclusion of light cycle, differences in sugar content between WT and transgenic plants persisted. A constantly low maltose content in the G1i transgenic lines was also detected (Fig. 6E).

**Figure 7:**
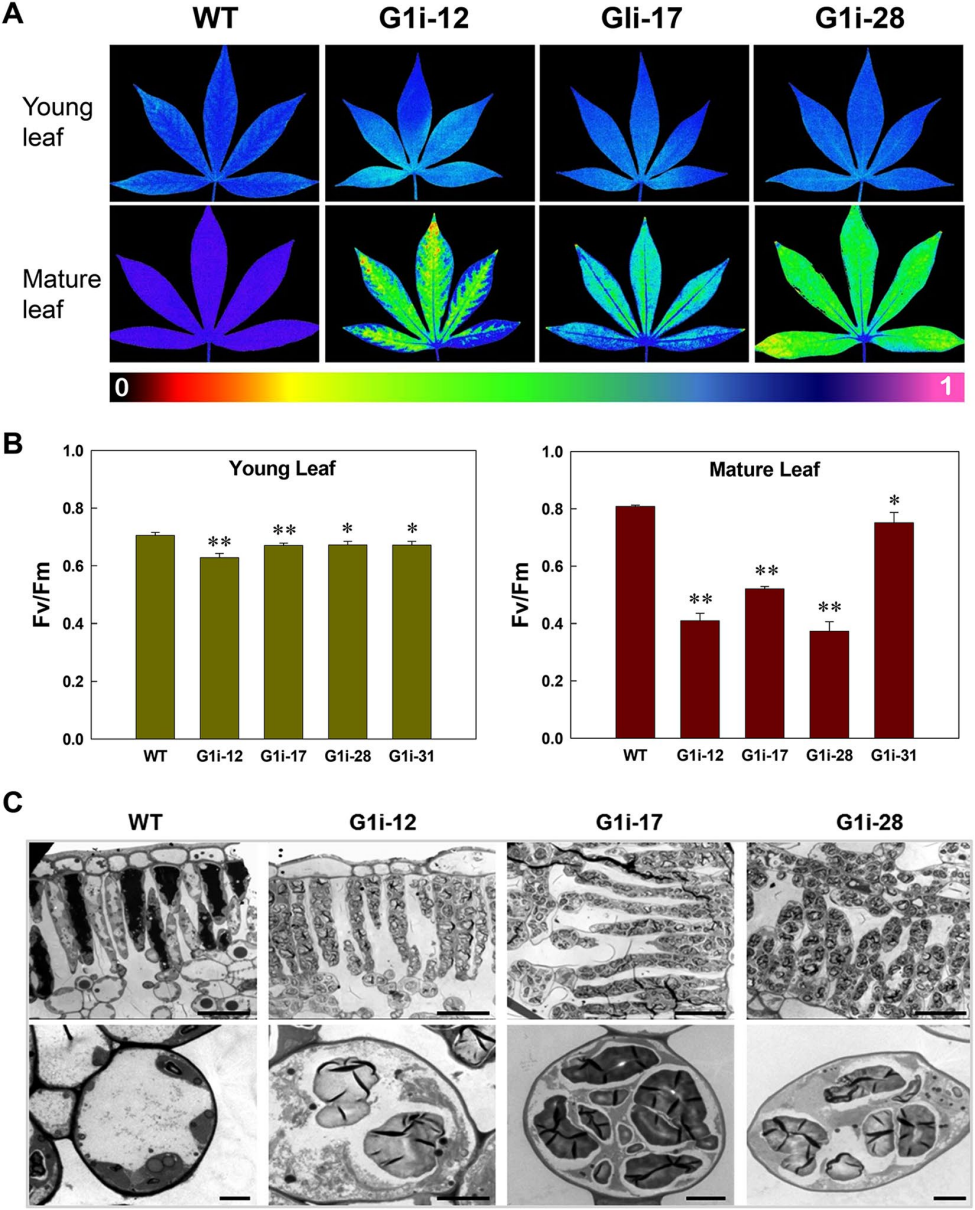
Photosynthetic parameters, mesophyll cells and chloroplasts of leaves from wild-type (WT) and G1i transgenic cassava. (**A**) F_v_/F_m_ changes of young and mature leaves. The color bar shows F_v_/F_m_ values from 0 to 1. (**B**) Quantification of F_v_/F_m_ from young and mature leaves. Values are presented as mean ± SD of three biological replicates. Significant differences were determined by the Student’s *t*-test: **P < 0.05; *P < 0.01. (**C**) Mesophyll cells (upper panel) and chloroplasts (lower panel) containing enlarged starch granules in the G1i transgenic cassava. Bars = 20 μm in the upper panel and 2 μm in the lower panel.

Photosynthetic capacity was reduced dramatically in the mature leaves of G1i transgenics when measured using an imaging chlorophyll fluorimeter. Quantitative analysis of maximum fluorescence ratio (F_v_/F_m_) also confirmed the dramatic decrease in the photosynthetic rate of transgenic mature leaves (Fig. 7A,B). Starch excess in mesophyll cells of G1i transgenic plants was also detected by TEM analysis (Fig. 7C) and their chloroplasts were replete with starch granules without a clear boundary of the chloroplast membrane (Fig. 7C).

### Repressed *MeGWD1* expression affects production of cassava storage roots

**Figure 8:**
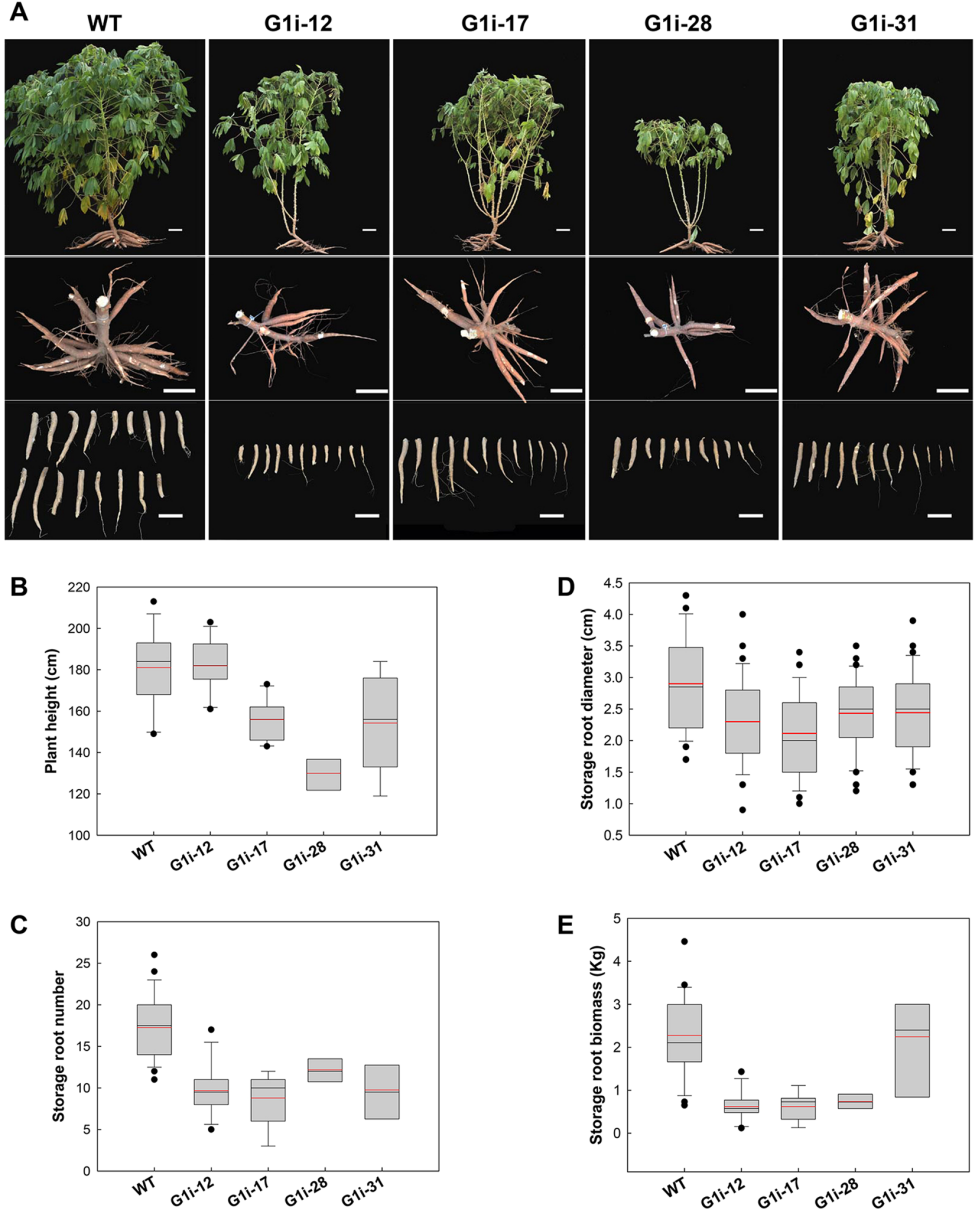
Phenotypic evaluation of wild-type (WT) and G1i transgenic cassava harvested from the field. (**A**) Canopy architecture (upper panel), attached storage roots (middle panel) and storage roots (lower panel) of harvested cassava plants from the field. Bars = 15 cm. (**B–E**) Storage root biomass (**B**, n > 6), storage root number (**C**, n > 6), storage root diameter (**D**, n > 10) and plant height (**E**, n > 6).

Under field growth conditions, reduced plant growth was observed in G1i transgenic plants, under both aerial and underground parts (Fig. 8). The height of G1i transgenic plants was lower than that of WT plants (Fig. 8A, upper panel; Fig. 8B). In contrast to the WT, which showed vigorous growth with green leaves and mature storage roots, G1i transgenic plants maintained fewer leaves. Their storage roots were also thinner than those of the WT plants (Fig. 8A, middle and lower panel). The average root diameter decreased to 2.1–2.4 cm as compared with 2.9 cm seen for WT plants and the average number was also markedly reduced: 9–12 roots/plant of transgenic lines as compared with 17 roots/plant for the WT plant (Fig. 8C,D). Under field conditions, the yield of WT storage roots was about 2.2 kg/plant. In addition, an average of a 30% reduction in root biomass of the G1i transgenic line was detected in these field-harvested plants (Fig. 8E).

**Figure 9:**
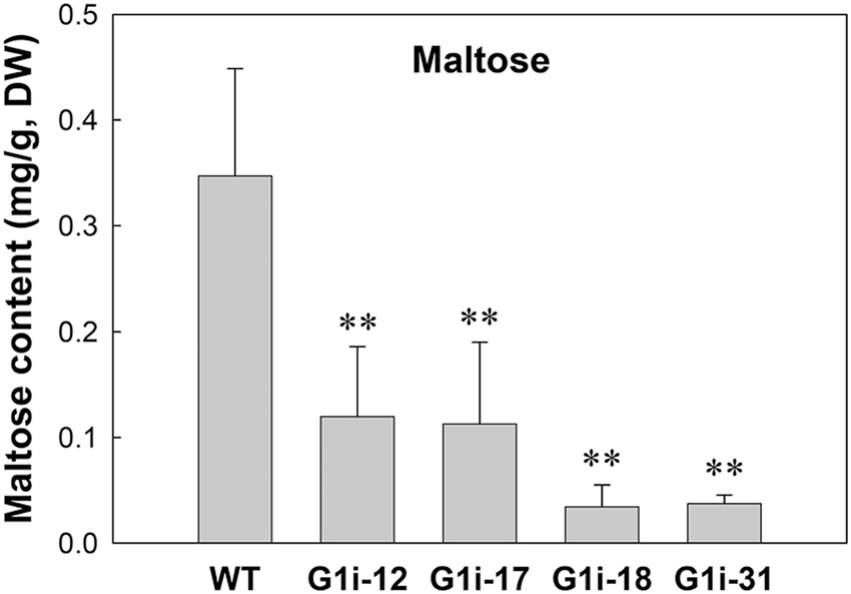
Maltose content of storage roots harvested from field-grown cassava. Values are presented as mean ± SD of three biological replicates. Significant differences were determined by the Student’s *t*-test (** *P* < 0.05).

To investigate the impact on sugar and starch metabolism in storage roots of G1i plants, the upper quartile, median and lower quartile of roots were selected and analyzed according to a root diameter boxplot (Fig. 8D, Supplementary Table 4). Similar to leaves, the maltose content decreased sharply in transgenic roots (Fig. 9). Moreover, no obvious trend was found for fructose, glucose and sucrose content, with the exception that the WSP content was slightly decreased in transgenic roots. The starch content was also significantly unaltered (Supplementary Fig. 8).

### GWD1 regulates starch degradation rate by β-amylase

**Figure 10:**
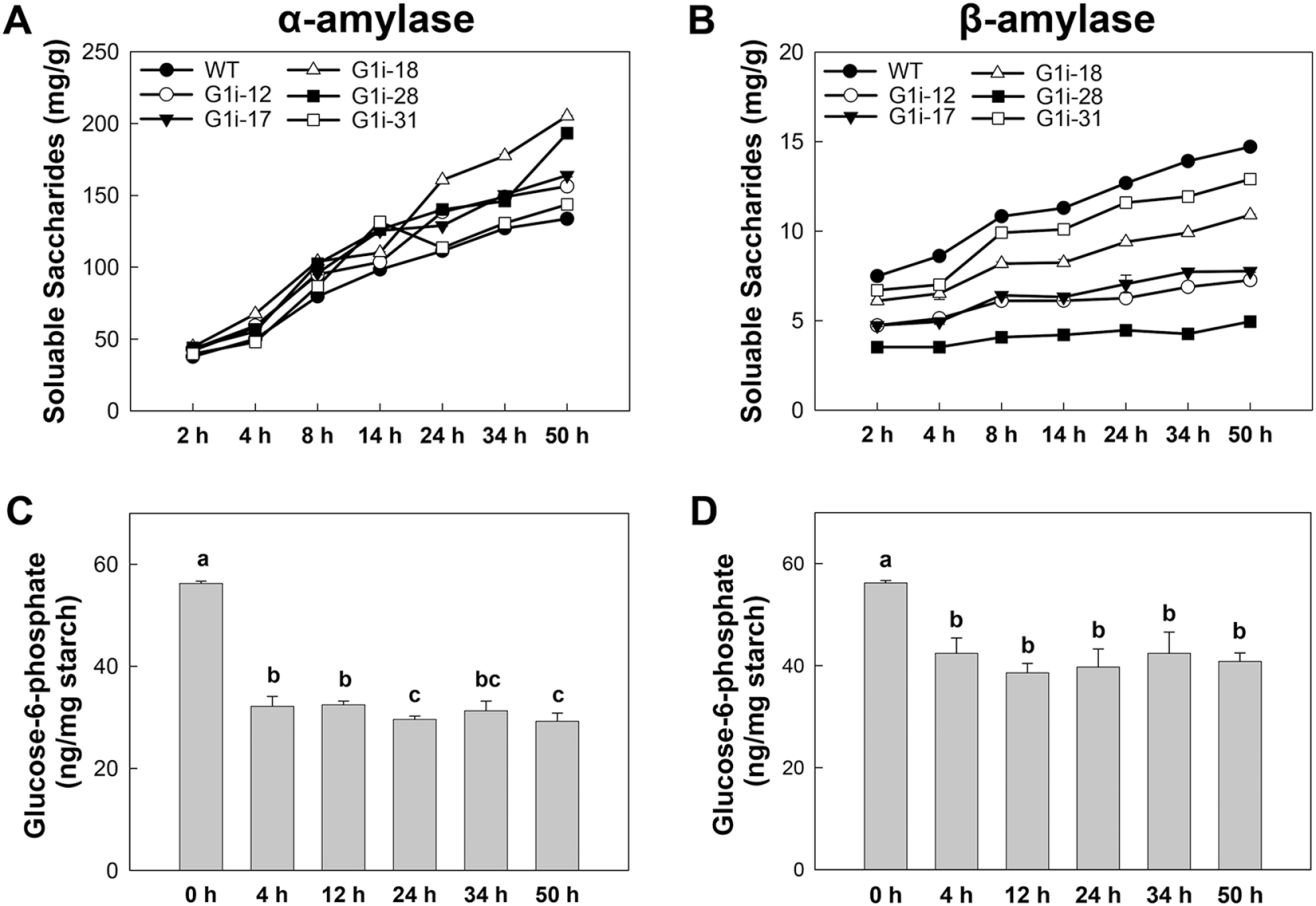
Amylolysis assay *in vitro* and phosphorylation level analysis of storage starches from wild-type (WT) and G1i transgenic cassava. (**A**,**B**) The α-amylolysis and β –amylolysis rates of storage starches are shown. (**C,D**) Altered phosphorylation levels in starches during treatment with α-amylase (**C**) and β-amylase (**D**) at different times. Values are presented as mean ± SD of three biological replicates. Values labeled with different letters (a, b, and c) were significantly different as determined by Duncan’s multiple comparison tests at an alpha value of *P* < 0.05.

To assess the effect of reduced starch phosphorylation on starch degradation, the native starch granules that were isolated from storage roots of the transgenic cassava and WT were treated with α-amylase and β-amylase, respectively. All starches from either G1i transgenic cassava or the WT showed a rapid increase in reducing sugar content following treatment, with a similar trend found for α-amylolysis (Fig. 10A). For WT starch, the levels of reducing sugar content reached about 200 mg/g starch after 50 h of α-amylase treatment as compared with only 15 mg/g starch following β-amylase treatment (Fig. 10B). For starches of G1i transgenic cassava, the β-amylolysis rate was further reduced as compared with that of the WT (Fig. 10B). For example, in the G1i-28 line, β-amylolysis was almost completely inhibited. Phosphate content of WT starches as determined by HPAEC-PAD revealed that after 4 h of treatment with α-amylase, the remaining phosphate content in the starch was reduced by 43% and only by 5% during the following 46 hours (Fig. 10C). For β-amylase treatment, the phosphorylation level was reduced by 25% during the first 4 hours and remained significantly unaltered during the following 46 hours of treatment (Fig. 10D).

### GWD1 plays a key role in maintaining transient starch morphogenesis

**Figure 11:**
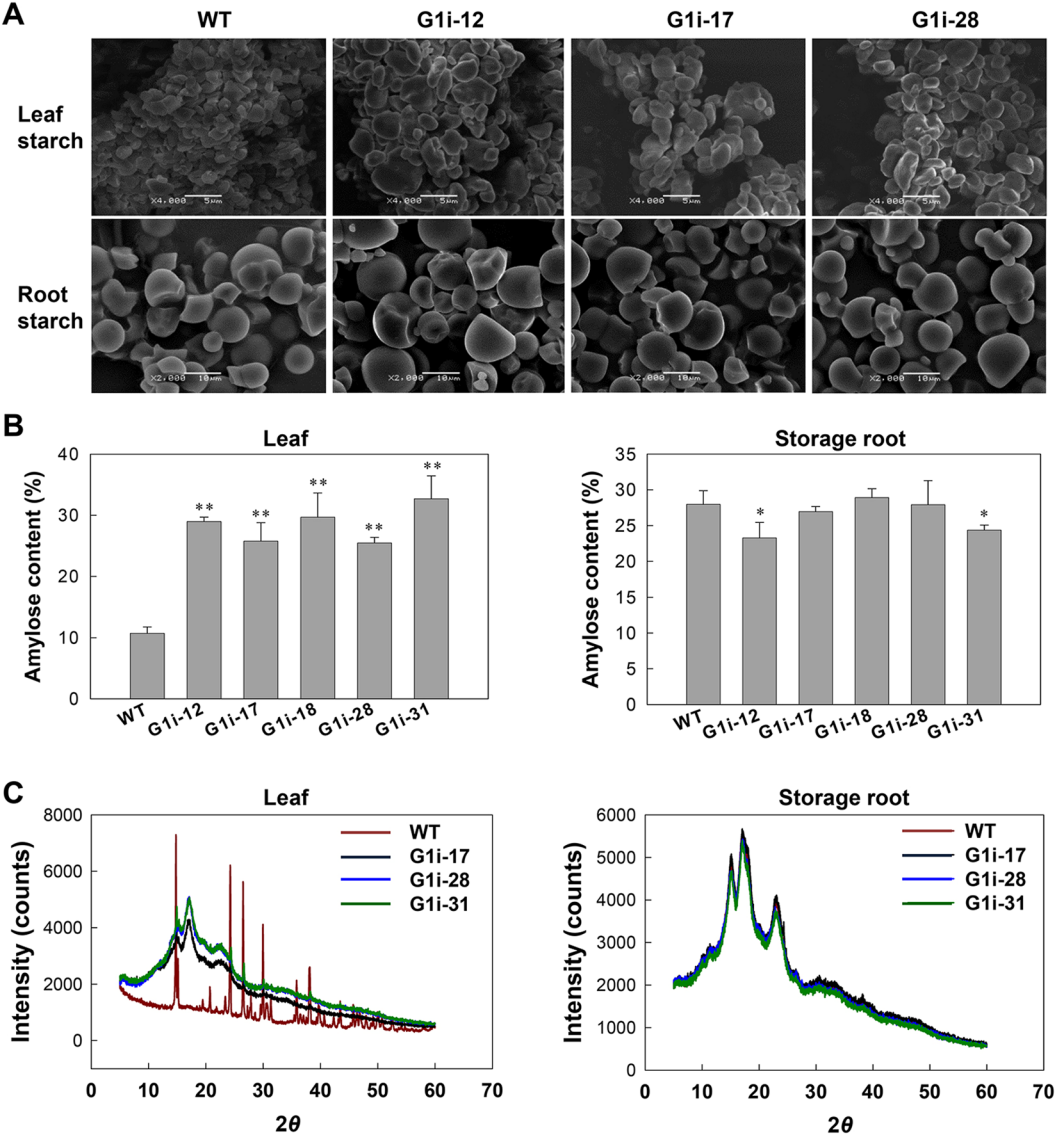
Morphology, amylose content, and X-ray powder diffraction spectra of starches in wild-type (WT) and G1i transgenic cassava. (**A**) Scanning electron micrographs of starch granules from mature leaves and storage roots at the end of the light phase. Bars = 5 μm in the upper panel and 10 μm in the lower panel. (**B**) Amylose content of mature leaf and storage root starches from WT and G1i transgenic cassava as analyzed by the colorimetric method. Values are presented as mean ± SD of three biological replicates. Significant differences were determined by the Student’s *t*-test: **P < 0.05; ***P < 0.01. (**C**) Diffractogram of transient starches that were isolated from mature leaves and storage starches from mature storage roots of WT and G1i transgenic cassava at the end of the light cycle.

Although morphological analysis of storage root starch by SEM analysis did not reveal any differences, the shape of transient starch found in the G1i lines was much greater and thicker (Fig. 11A). The maximal diameter of G1i transient starch granules was 10 μm while that for WT granules was 3 μm. The WT’s transient starch displayed a homogenous population of granules with a round and flat discoid shape, which is a typical structural appearance of starch granules that is found in leaves^23, 34^. By contrast, transient starch granules of G1i transgenic lines were more heterogeneous (Fig. 11A).

Amylose content analysis revealed that the transient starch content of G1i transgenic leaves increased dramatically to about 33% as compared with 11% found in WT plants (Fig. 11B). However, no obvious change in amylose content in storage starch was observed between G1i (that ranged from 23–29%) and WT (27%). Wide-angle X-ray scattering (WXRD) showed that the WT transient starch displayed high crystallization (Fig. 11C, left panel). Interestingly, the diffraction pattern of transient starch of G1i lines showed A-type crystalline starch, which was similar to storage starches (Fig. 11C). No change was found in the storage root of WT and G1i transgenic cassava plants (Fig. 11C, right panel).

The chain length distribution profiles by HPAEC-PAD for native starches from each line were compared (Supplementary Fig. 9). Transient starches of WT plants showed two peaks at DP 13–14 and around DP 45, and a shoulder at DP 20, which was similar to that of storage starches from potato, maize and sweet potato^35, 36^. However, a trough at DP 8, usually exists in other species, and was not detected for transient starch of WT plants, which resembled cassava storage starch. Compared with the WT, transient starches of transgenic lines showed a higher proportion of short chains (DP 6–13), fewer median chains (DP 15–35) and slightly higher long chains (DP 37–48) (Supplementary Fig. 9A). Interestingly, the X-ray diffraction pattern of transgenic transient starches is similar to that of cassava storage root starches. Furthermore, transient starch granules that were harvested from transgenic plant leaves showed irregular “zebra stripes” (Fig. 7C) which was also similar to starches derived from storage roots as visualized by TEM^37^. The findings indicated that the leaf transient starches of G1i transgenic plants were transformed to “storage starch”. No significant difference was found when comparing root starches between WT and G1i transgenic lines (Supplementary Fig. 9B).

Using differential scanning calorimetry, the thermal stability of starch granules was analyzed (Tables 1, and 2). The onset temperature (T_o_) of transient starches of G1i transgenic lines was unchanged when compared to WT transient starch; however, the peak temperature (T_p_) and conclusion temperature (T_c_) increased slightly. Moreover, their enthalpy had increased to 11–13 J/g, which was much higher than that found in the WT (3.1 J/g, Table 1). The present findings are in agreement with previously reported observation^38^.

**Table 1:** Differential scanning calorimetry (DSC) of thermal properties in transient starch from wild type (WT) and G1i transgenic cassava leaves.

Leaf starch	T_o_ (°C)	T_p_ (°C)	T_c_ (°C)	ΔH (J/g)
WT	64.1 ± 0.07^bc^	66.9 ± 0.09^e^	69.7 ± 1.01^b^	3.1 ± 0.39^d^
G1i-12	64.8 ± 0.15^a^	67.9 ± 0.25^a^	71.5 ± 0.40^a^	11.1 ± 0.58^c^
G1i-17	64.9 ± 0.09^a^	67.7 ± 0.19^ab^	71.2 ± 0.19^a^	12.8 ± 0.61^a^
G1i-18	63.99 ± 0.07^c^	67.3 ± 0.19^cd^	71 ± 0.26^a^	12.4 ± 0.36^ab^
G1i-28	64.8 ± 0.12^a^	67.5 ± 0.16^bc^	71.3 ± 0.22^a^	13.1 ± 0.81^a^
G1i-31	64.2 ± 0.19^b^	67.2 ± 0.13^de^	71 ± 0.38^a^	11.7 ± 0.34^bc^

Note: Values represent the mean ± SD of three biological replicates. Values labeled with different letters are significantly different by Duncan’s multiple comparison tests at *P* < 0.05.

**Table 2:** Differential scanning calorimetry (DSC) of thermal properties in storage starch from storage roots of wild type (WT) and G1i transgenic cassava.

Root starch	T_o_ (°C)	T_p_ (°C)	T_c_ (°C)	ΔH (J/g)
WT	57 ± 0.13^b^	60.1 ± 0.16^b^	76.5 ± 1.04^b^	11.9 ± 1.13^ab^
G1i-12	55.6 ± 0.29^c^	59.4 ± 0.36^b^	80 ± 0.93^a^	12.9 ± 0.45^a^
G1i-17	57.7 ± 0.03^a^	62.7 ± 1.39^a^	78.4 ± 0.27^ab^	9.5 ± 0.23 ^cd^
G1i-18	55 ± 0.07^d^	58.6 ± 0.14^c^	77.5 ± 2.03^b^	13.6 ± 1.22^a^
G1i-28	54.6 ± 0.07^e^	58.2 ± 0.32^c^	64.7 ± 0.22^d^	7.8 ± 0.38^d^
G1i-31	56.8 ± 0.18^b^	60 ± 0.15^b^	74.1 ± 1.74^c^	10.9 ± 1.55^bc^

Note: Values represent the mean ± SD of three biological replicates. Values labeled with different letters are significantly different by Duncan’s multiple comparison tests at *P* < 0.05.

## Discussion

The development of storage roots in root crops is a biological process that sustains efficient accumulation of starch and other health-promoting components including anthocyanins and carotenes^3, 5, 7^. However, the molecular mechanism underlying storage root development and regulation, especially photo-assimilation from source, is largely unknown. Mobilization of carbon flux toward starch biosynthesis, either in the context of altered glycolysis/gluconeogenesis activity during storage root development^7^ or the transition of carbon flux from phenylpropanoid biosynthesis to carbohydrate metabolism and starch biosynthesis during domestication^39^, have been suggested in cassava but still lack direct evidence supporting such an assertion. In the potato, starch mobilization from leaves to tubers could be affected by reduced levels of starch phosphorylation due to repressed GWD1 expression^18^. In this study, we showed that GWD1 regulated starch phosphorylation is essential for transient starch morphogenesis and turnover in cassava leaves and empirically contributes to source/sink strength, thus, affecting storage root growth in the cassava. This mechanism contributes to the intrinsic source-sink relationship in the root crop.

In leaves, starch phosphorylation is essential for normal starch metabolism, leading to increased sugar content in the light and decreased sugar content in the dark as confirmed in various plants like Arabidopsis^40^, *Lotus japonicas* ^41^, potato^12, 18^ and maize^16^. Cassava GWDs showed similar diurnal expression patterns with many genes involved in starch metabolism and consistent with starch content oscillation in cassava leaves (Fig. 3). Putative light regulatory elements were also found in the *MeGWD1* promoter such as the G-box and MYB binding site^42^, indicating that GWD expression is strictly regulated by light signals. Moreover, consistent expression of GWDs in storage roots during the day/night cycle and upregulation in the developing storage roots imply important roles in cassava storage roots. Normally the amount of GWD1 protein is very low in plants. Even a weak reduction in transcription might lead to expression levels being less than the limits of detection by Western blot analysis of G1i transgenic plants. In G1i transgenic cassava, the inhibition of starch turnover and the phenotype of starch excess were consistent with that of GWD1 mutants commonly found in other species. The starch excess resulted in a negative feedback effect on photosynthesis, leading to a reduced supply of photosynthetic products in the source and senescence phenotype^34, 43^. Reduced sugar levels, especially of sucrose and maltose in Gli transgenic cassava indicate a decreased source capacity for carbohydrate efflux. Consequently, starch accumulation in young stems and storage root growth was affected as evidenced in the *srd* mutant and G1i transgenic cassava plants. Due to the higher repression of GWD1 expression in G1i transgenic cassava lines than is found in the *srd* mutant, a strong negative effect on storage root development was observed. In GWD1 knock-out *sex1* Arabidopsis, reduced biomass was reported^44^. Transgenic potato with anti-sense inhibition of GWD1 (R1) expression constitutively increased amylose content without altering the phenotype^18^. Silencing GWD1 expression by RNAi in monocots like maize or rice also led to an excess of leaf starch without negatively impacting biomass or growth rate^16, 24^. Thus, our results provided direct evidence of the role of GWD1 in cassava storage root development by regulating transient starch turnover and carbohydrate partitioning.

Several studies have previously reported *in vivo* leaf starch degradation that is catalyzed by β-amylase^17, 45^. In the cereal endosperm, ɑ-amylase initiates starch degradation^10^. In cassava, the rapid decrease in phosphorylation in amylolysis assay indicates that starch phosphorylation was necessary for both α- and β-amylases. However, starch degradation by β-amylase was greatly affected by the starch phosphorylation level, unlike α-amylase (Fig. 10). Significant reductions in maltose in G1i transgenic leaves in the dark cycle also suggests that cassava transient starch was mainly degraded by β-amylase (Fig. 6). In brief, starch turnover in cassava leaves required the collaboration of GWD1 and β-amylase rather than α-amylase^13^. Metabolically, it is rational for plants to use GWD1/β-amylase as a primary starch degradation pathway. It helps regulate the rate of starch degradation by altering the level of starch phosphorylation, and does so to avoid carbohydrate starvation and adapt rapidly to physiological and environmental stresses; for example in the context of cold climatological conditions^46, 47^.

In addition to maltose, leaves of the G1i transgenic cassava also displayed reduced levels of other sugars during the whole light/dark rhythm (Fig. 6), unlike that found in other starch-excess plant mutants^48, 49^. Since cellular sugars like maltose, glucose, sucrose and WSP provide carbon sources and affect the growth, development and metabolism of plants by activating signaling or by regulating gene expression^34, 49, 50^, the lower sugar content indicates reduced carbon availability. In particular, reduced sucrose metabolism might also affect sugar signaling, thereby regulating metabolic processes by modulating the expression of transcriptional factors and microRNAs^51, 52^. Therefore, GWD1 is pivotal for maintaining sugar homeostasis in association with β-amylase in cassava leaves.

The decrease in transient starch phosphorylation in G1i transgenic cassava results in dramatic changes in starch structure. Morphologically, transient starch granules of G1i transgenic cassava were much bigger and displayed heterologous size distributions as compared with that of their WT counterparts. Amylose content of the transient starch of G1i transgenic cassava increased significantly up to 33% which is also close to the amylose content of cassava storage starch^36^. Interestingly, X-ray diffraction images indicated that transient starches from G1i transgenic cassava displayed a typical type A crystal structure similar to storage starch^37^ (Fig. 7C), which differs from the highly crystalline transient starch of WT (Fig. 11C). The transient starch displayed “zebra stripes” in TEM images, which is characteristic of storage starch in cassava^37^. Further, similar profiles of chain length distribution and gelatinization enthalpy between transgenic transient starches and WT storage starch indicates a transformation of transient starch to storage starch in the G1i cassava leaves. Such dramatic changes occurred with an increased amylose content in transient starch, which was affected by reduced functional activity of GWD1^23, 34^. The transformation indicates that the level of GWD1 might hold the key to control transient or storage starch morphogenesis in different organs.

Field evaluation of G1i transgenic cassava showed reduced growth vigor. In addition, root biomass was decreased by fewer storage roots and reduced size, which indicated that initiation and expansion of the storage root was markedly repressed. Such phenotypic changes illustrate the effect of source strength on storage root development. In the WT, the phosphorylation level of storage starch in cassava roots (57 ng/mg starch) was more than two-fold that of transient starch from cassava leaves. The significance of higher phosphorylation level of root starch still needs to be determined. HPAEC analysis also revealed a sharply reduced glucose-3-phosphate in the G1i transgenic lines, suggesting that reduced phosphorylation at C3 position was due to the absence of phosphoglucan substitute required for GWD3^32, 53, 54^. However, with the exception of the drastic decrease in phosphate levels, the starch in G1i storage roots did not significantly change its physico-chemical properties, including the amylose content, thermal parameters and chain length distribution. During storage root formation, the increased GWD1 expression in developing storage roots indicates its role in starch degradation by providing sufficient sugars to support the sink expansion. In harvested mature storage roots, the *MeGWD1* expression was up-regulated again during the post-harvest storage, which suggested that *MeGWD1* was involved in starch degradation during post-harvest physiological deterioration.

In summary, this study unraveled *MeGWD1* function in the cassava, and especially linked source strength and storage root growth. Normally, transient starch is mobilized by synergistic interactions between GWD and β-amylase in dark phases of the day/light cycle, which provides source of carbon for leaf metabolism and sucrose synthesis that is then exported to storage roots to support storage root development. Carbohydrates that are imported from the source sustain expansion and starch accumulation in the cassava storage root. In its early stage of development, MeGWD1 might also mediate starch mobilization to support faster growth. When the storage roots reach maturity, cassava reduces the expression of MeGWD1 to facilitate storage starch granule formation. Overall, our study has revealed the role of GWD1 in root crops by affecting transient starch morphogenesis and a source/sink relationship that has not been previously reported.

## Methods

### Identification of *srd* cassava mutant

The *srd* mutant was obtained from a batch of cassava T-DNA lines growing at SIPPE greenhouse, Shanghai, China. The T-DNA lines were transgenic cassava (cultivar TMS60444) that was integrated with T-DNA harboring a CaMV 35S::HPT expression cassette. After 3 months of growth, the potted plants were harvested to screen for the production of storage roots and subsequent molecular and physiological analysis. Their stem cuttings were planted in pots in the greenhouse for macropropagation and growth evaluation.

### Inverse PCR analysis of the T-DNA flanking sequence

The T-DNA flanking region was isolated by inverse-nested PCR using the inverse primer designed from the 35S promoter and hygromycin sequences as described in Uchiyama and Watanabe^55^. The PCR products were purified, cloned into the pEASY- Blunt Simple vector (Transgen Biotech, Beijing, China) and sequenced.

### Cloning of GWD genes and phylogenetic tree analysis

The sequence information of GWDs were derived from cassava full-length cDNA clones database (RIKEN, Japan; http://www.brc.riken.jp/inf/en/DB/) and cassava genome database (http://www.phytozome.net). The full length cDNAs of *MeGWD1*, *MeGWD2* and *MeGWD3* were cloned from a cassava cDNA library and the resulting sequences were submitted to the NCBI database (Genbank Accession Numbers: JN618458, JN618459 and JN618460, respectively). The GWD protein sequences from various plant species were collected from the NCBI database (cutoff e^−4^). Protein sequence alignment was performed with the ClustalX package and submitted to MEGA 5.0 software to generate a branched phylogenetic tree^56, 57^. The neighbor-joining method with 1000 replicates of a bootstrap analysis was used to assess the statistical reliability of the tree topology.

### Production of *MeGWD1* RNAi transgenic cassava and Southern blot analysis

The binary expression vector p35S::GWD1RNAi was constructed on the basis of the plasmid pRNAi-dsAC1^58^. The AC1 sequence was replaced with a partial cDNA sequence of *MeGWD1* from 741 bp to 1193 bp. The resulting construct was introduced into *Agrobacterium tumefaciens* LBA4404 and the cassava cultivar TMS60444 was used to generate transgenic plants as described previously by Zhang *et al*.^59^. The integration pattern of T-DNA in transgenic cassava was performed according to the standard protocol of Southern blot analysis as described by Zhao *et al*.^37^. The DIG-labeled hygromycin phosphotransferase gene (HPT) gene was used as probe for hybridization.

Transgenic lines (referred to as G1i for short) and wild type (WT) were propagated *in vitro* and then transferred to pots in the greenhouse for macropropagation (16 h/8 h of light/dark, 30 °C/22 °C day/night). For field evaluation, 10 stems per transgenic line and WT were planted in early May in the Wushe Plantation for Transgenic Crops, Shanghai, China (31°13948.0099 N, 121°28912.0099E), and harvested in early November. The performance of plants was phenotypically recorded.

### Gene expression analysis by qRT-PCR and Western blot

Leaves from at least three plants per line were harvested and then ground in liquid N_2_ for mRNA extraction. To quantify the expression of *GWD* genes, qRT-PCR was performed as described^60^. β-Actin was used as a reference for normalization. The primers were as follows:

*MeGWD1* (forward, 5′- ACCTCTGCATGGCTGTCCTGGTT-3′; reverse, 5′-GCACGGCCGGGATAGGCTCC-3′), *MeGWD2* (forward, 5′-CATGGCTGTGCTGATTCAAG-3′; reverse, 5′-GAAGCTCATTGCTCGTCCTG-3′), *MeGWD3* (forward, 5′-GCCGTCGAGCTGCTGGTGTGT-3′; reverse, 5′-CGCCAAGGAGTGCCCCGAGT-3′), and *β-Actin* (forward, 5′-TGATGAGTCTGGTCCATCCA-3′; reverse, 5′-CCTCCTACGACCCAATCTCA-3′).

The qRT-PCR analysis of GWD1 expression in *srd* mutant was performed using the specific primers (G1qrt5′F, 5′-CACAAAGCAAGATACACGCTC-3′; G1qrt5′R, 5′-ACAAGCTGTGCTAAGTCCCT-3′) located at 5′ end of insertion region. The GWD1 protein in cassava leaves was analyzed using Western blot with an antibody directed against MeGWD1. Total protein was extracted from leaves as described by Ritte *et al*.^61^. Protein was quantified according to the method of Bradford^62^. Approximately 60 μg of protein was loaded on an SDS-PAGE and immuno-blotting was performed with rabbit anti-MeGWD1 antiserum.

### Sugar and starch content analysis

Leaves and roots of six-month-old transgenic and WT cassava were harvested from three independent plants and then baked at 80 °C for two days to obtain a constant dry weight (DW). The same type of samples per plant line were mixed and ground together, respectively. The dried sample (30 mg) was dissolved in 0.7 ml of 80% ethanol, thoroughly vortexed and then incubated at 70 °C for 2 h. Aliquots of 0.7 ml HPLC-grade water and 0.7 ml chloroform were added to the sample. After shaking several times, the mixtures were centrifuged at 12000 g for 10 min. The pellet was collected for starch content analysis while the supernatant was composed of soluble sugar. The starch pellet was washed three times with 80% ethanol and total starch content was analyzed using total starch kit (Megazyme International Ireland Limited, Wicklow, Ireland). The aqueous supernatant of 0.7 ml was transferred into a 1.5-ml micro-centrifuge tube and mixed with 0.7 ml chloroform. After centrifugation at 12000 g for 10 min, 0.5 ml of the supernatant was transferred to a glass tube for HPLC analysis of each sugar component. The sugar-separation method that was used was slightly modified from the original protocol described by the manufacturer; the Agilent technologies HPLC column (ZORBAX Carbohydrate column; 4.6 × 150 mm, 5 μm) with a differential refraction detector was then used. The mobile phase consisted of 75% acetonitrile with a flow rate of 0.8 ml/min and the temperature of the column was maintained at 35 °C. The sugar types were identified based on retention time of the standards, and the sample concentration was calculated from the external standard curve. Data was calculated from three biological replicates.

### Determination of chlorophyll fluorescence

Leaves were collected from the same position of three-month-old plants in the greenhouse. Prior to detection, leaves were adapted to darkness for 30 min. The photosynthetic activity was recorded via chlorophyll fluorescence determinations of the photochemical yield (F_v_/F_m_), which represented the maximum quantum yield of PSII, using an imaging chlorophyll fluorimeter (Walz Imaging PAM, Walz GmbH, Effeltrich, Germany). The measurements were conducted at room temperature (25 °C) using standard saturated light mode. The actinic light intensity was 10 µmol m^−2^ s^−1^. The F_v_/F_m_ value was analyzed using ImagingWin Software. Leaves from three independent plants per line were used and data was calculated from three biological replicates.

### Iodine staining, starch extraction and amylase assay

The leaves of three-month-old cassava plants from the greenhouse were submerged in 80% ethanol and incubated at 37 °C for 8 h with vigorous shaking to remove chlorophyll. The staining was performed using 0.5 × Lugol’s dye for 30 min. The excess Lugol’s stain was removed by washing with distilled water. Cross sections of stems were cut transversally and longitudinally before staining with Lugol’s dye.

Mature leaves (10–15 g) were harvested form at least three cassava plants per line in field and homogenized in liquid nitrogen. Transient starch in the homogenate was extracted and purified using 95% percoll^61^. The storage starch was extracted from storage roots that were harvested from six-month-old cassava plants grown in the field and washed twice in 75% ethanol and air-dried overnight at 40 °C^37^.

The α-amylase and β-amylase were purchased from Sigma (A4582, A7005). A 1% native starch suspension was prepared using 1.0 M sodium citrate (pH 5.6). One unit of amylase was defined as the amount of enzyme that liberated 1 mg reducing sugar as maltose per 5 min with soluble starch substrate at 40 °C. Ten units of amylase were added into 10 ml of a 1% starch suspension and incubated at 40 °C. Each 1 ml of suspension was collected at 2, 4, 8, 14, 24, 34, and 50 h and then centrifuged. The reducing sugar of the aqueous fraction was analyzed using the DNS method. Maltose was used as a standard curve. The pellet was washed three times using 80% ethanol. The remaining phosphate of starch granules was analyzed using HPAEC-PAD after acid hydrolysis. Data was calculated from three biological replicates.

### Physicochemical property assay of transient and storage starches

The starch phosphorylation and chain length distribution were carried out following the protocol previously described by Ritte *et al*.^61^ using high performance anion exchange chromatography (HPAEC) that was equipped with a CarboPac PA1 column. Starch was hydrolyzed in 0.7 N HCl before determining the phosphorylation level. The chain length distribution of amylopectin was started by boiling 5 mg starch and enzymatic digestion with isoamylase (I5282, Sigma, St. Louis, MO, USA). Oligosaccharides with polymerization degree 4–7 (47265, Sigma, St. Louis, MO, USA) were used as a standard.

X-ray diffraction analysis of the extracted starch powder was performed using a D8 Advance Bruker X-ray diffractometer (Bruker AXS, Karlsruhe, Germany) and scanned through the 2*θ* range of 5–60° at a rate of 4° min^−1^. The thermal properties of the starch samples were analyzed using a differential scanning calorimeter (DSC, Q2000; TA Instruments Ltd., Crawley, UK) at a temperature scanning range of 30–95 °C. The gelatinization temperature and enthalpy were determined using the Universal Analysis 2000 (TA Instruments Ltd., Crawley, UK). The amylose content was measured using the colorimetric amylose content determination as described in Knutson and Grove^63^. Amylose type III and amylopectin (Sigma A0512 and Sigma 10118, St. Louis, MO, USA) from potato were used to establish a standard curve.

### Scanning electron microscopy and transmission electron microscopy

Isolated starch granules in distilled water were dropped and sprinkled on double-sided sticky tape, air-dried, and coated with gold powder. Samples were observed and photographed by scanning electron microscopy (SEM, JSM6360lv, JEOL, Tokyo, Japan). Pieces of fresh mature leaves (2 mm^3^) were coated with 1,3-diformal propane and subjected to ultra-thin sectioning. The sections were photographed under a transmission electron microscope (TEM; Hitachi H7650, Tokyo, Japan).

### Statistical analysis

Basically, the leaf and root samples were collected from three independent plants per line and then mixed together for further analyses. Data from at least three biological replicates were presented as mean ± SD. Analysis of variance (ANOVA) by Duncan’s multiple comparison tests or independent samples Student’s *t*-test was performed using SPSS software, version 17 (SPSS Inc., Chicago, IL, USA). An alpha value of *P* < 0.05 was considered statistically significant.

## Electronic supplementary material

Supplementary Information

## References

[1] Ceballos, H., Hershey, C. & Becerra-López-Lavalle, L. A. New approaches to cassava breeding. In *Plant Breeding Reviews*, Vol. 36 (ed. Janick, J.), John Wiley & Sons, Inc., Hoboken, NJ, USA: p.427–504 (2012).

[2] Liu, Q., Liu, J., Zhang, P. & He, S. Root and tuber crops. In *Encyclopedia of Agriculture and Food Systems*, Vol. 5 (ed. Alfen N.V.), Elsevier San Diego: p. 46–61 (2014).

[3] El-Sharkawy MA (2004). Cassava biology and physiology. Plant Mol. Biol..

[4] Okogbenin E (2013). Phenotypic approaches to drought in cassava: review. Front. Physiol..

[5] Alves, A. A. C. Cassava botany and physiology. In *Cassava: Biology, Production and Utilization* (ed Hollocks, R. J. & Thresh, J. M.), CAB international; p. 67–89 (2002).

[6] Pelacani CR, Cruz JL, Mosquim PR (2003). Carbon partitioning and assimilation as affected by nitrogen deficiency in cassava. Photosynthetica.

[7] Yang J, An D, Zhang P (2011). Expression profiling of cassava storage roots reveals an active process of glycolysis/gluconeogenesis. J. Integr. Plant Biol..

[8] Pellet D, El-Sharkawy MA (1994). Sink-source relations in cassava: effects of reciprocal grafting on yield and leaf photosynthesis. Exp. Agr..

[9] Smith AM, Stitt M (2007). Coordination of carbon supply and plant growth. Plant Cell Environ..

[10] Smith AM, Zeeman SC, Smith SM (2005). Starch degradation. Annu. Rev. Plant Biol..

[11] Skeffington AW, Graf A, Duxbury Z, Gruissem W, Smith AM (2014). Glucan, water dikinase exerts little control over starch degradation in Arabidopsis leaves at night. Plant Physiol..

[12] Yu TS (2001). The Arabidopsis sex1 mutant is defective in the R1 protein, a general regulator of starch degradation in plants, and not in the chloroplast hexose transporter. Plant Cell.

[13] Kötting O (2009). STARCH-EXCESS4 is a laforin-like phosphoglucan phosphatase required for starch degradation in *Arabidopsis thaliana*. Plant Cell.

[14] Stitt M, Zeeman SC (2012). Starch turnover: pathways, regulation and role in growth. Curr. Opin. Plant Biol..

[15] Ral JP (2012). Down-regulation of glucan, water-dikinase activity in wheat endosperm increases vegetative biomass and yield. Plant Biotechnol. J..

[16] Weise SE (2012). Engineering starch accumulation by manipulation of phosphate metabolism of starch. Plant Biotechnol. J..

[17] Blennow, A. Phosphorylation of the starch granule. In *Starch: Metabolism and Structure* (ed. Nakamura, Y.), Springer Japan; p. 399–424 (2015).

[18] Lorberth R, Ritte G, Willmitzer L, Kossmann J (1998). Inhibition of a starch-granule-bound protein leads to modified starch and repression of cold sweetening. Nat. Biotechnol..

[19] Carpenter MA (2015). Starch phosphorylation in potato tubers is influenced by allelic variation in the genes encoding glucan water dikinase, starch branching enzymes I and II, and starch synthase III. Front. Plant Sci..

[20] Shaik SS (2016). Starch granule re-structuring by starch branching enzyme and glucan water dikinase modulation affects caryopsis physiology and metabolism. PLoS ONE.

[21] Nielsen TH, Wischmann B, Enevoldsen K, Moller BL (1994). Starch phosphorylation in potato-tubers proceeds concurrently with *de novo* biosynthesis of starch. Plant Physiol..

[22] Wischmann B, Nielsen TH, Moller BL (1999). *In vitro* biosynthesis of phosphorylated starch in intact potato amyloplasts. Plant Physiol..

[23] Mahlow S (2014). Phosphorylation of transitory starch by α-glucan, water dikinase during starch turnover affects the surface properties and morphology of starch granules. New Phytol..

[24] Hirose T (2013). Disruption of a rice gene for a-glucan water dikinase, OsGWD1, leads to hyperaccumulation of starch in leaves but exhibits limited effects on growth. Front. Plant Sci..

[25] Bowerman AF (2016). Suppression of glucan, water dikinase in the endosperm alters wheat grain properties, germination and coleoptile growth. Plant Biotechnol. J..

[26] Cruz JL, Mosquim PR, Pelacani CR, Araujo WL, DaMatta FM (2003). Carbon partitioning and assimilation as affected by nitrogen deficiency in cassava. Photosynthetica.

[27] Sakurai T (2007). Sequencing analysis of 20,000 full-length cdna clones from cassava reveals lineage specific expansions in gene families related to stress response. BMC Plant Biol..

[28] Emanuelsson O, Nielsen H, Von Heijne G (1999). ChloroP, a neural network-based method for predicting chloroplast transit peptides and their cleavage sites. Protein Sci..

[29] Ritte G (2002). The starch-related R1 protein is an alpha -glucan, water dikinase. Proc. Natl. Acad. Sci. USA.

[30] Mikkelsen R, Suszkiewicz K, Blennow A (2006). A novel type carbohydrate-binding module identified in alpha-glucan, water dikinases is specific for regulated plastidial starch metabolism. Biochemistry.

[31] Christiansen C (2009). A CBM20 low-affinity starch-binding domain from glucan, water dikinase. FEBS Lett..

[32] Mikkelsen R, Blennow A (2005). Functional domain organization of the potato alpha-glucan, water dikinase (GWD): evidence for separate site catalysis as revealed by limited proteolysis and deletion mutants. Biochem. J..

[33] Smith SM (2004). Diurnal changes in the transcriptome encoding enzymes of starch metabolism provide evidence for both transcriptional and posttranscriptional regulation of starch metabolism in arabidopsis leaves. Plant Physiol..

[34] Stettler M (2009). Blocking the metabolism of starch breakdown products in Arabidopsis leaves triggers chloroplast degradation. Mol. Plant.

[35] Zhou W (2015). Impact of amylose content on starch physicochemical properties in transgenic sweet potato. Carbohyd. Polym..

[36] Rolland-Sabaté A (2012). Structural characterization of novel cassava starches with low and high-amylose contents in comparison with other commercial sources. Food Hydrocol..

[37] Zhao SS, Dufour D, Sanchez T, Ceballos H, Zhang P (2011). Development of waxy cassava with different biological and physico-chemical characteristics of starches for industrial applications. Biotechnol. Bioeng..

[38] Moorthy SN (2002). Physicochemical and functional properties of tropical tuber starches: a review. Starch/Stärke.

[39] Wang W (2014). Cassava genome from a wild ancestor to cultivated varieties. Nat. Commun..

[40] Baunsgaard L (2005). A novel isoform of glucan, water dikinase phosphorylates pre-phosphorylated alpha-glucans and is involved in starch degradation in Arabidopsis. Plant J..

[41] Vriet C, Smith AM, Wang TL (2014). Root starch reserves are necessary for vigorous re-growth following cutting back in *Lotus japonicus*. PLoS ONE.

[42] Gangappa SN, Maurya JP, Yadav V, Chattopadhyay S (2013). The regulation of the Z- and G-Box containing promoters by light signaling components, SPA1 and MYC2, in Arabidopsis. PLoS ONE.

[43] Zeeman SC, Rees TA (1999). Changes in carbohydrate metabolism and assimilate export in starch‐excess mutants of Arabidopsis. Plant Cell Environ..

[44] Caspar T (1991). Mutants of Arabidopsis with altered regulation of starch degradation. Plant Physiol..

[45] Edner C (2007). Glucan, water dikinase activity stimulates breakdown of starch granules by plastidial β-amylases. Plant Physiol..

[46] Sicher R (2011). Carbon partitioning and the impact of starch deficiency on the initial response of Arabidopsis to chilling temperatures. Plant Sci..

[47] An D, Yang J, Zhang P (2012). Transcriptome profiling of low temperature-treated cassava apical shoots showed dynamic responses of tropical plant to cold stress. BMC Genomics.

[48] Chia T (2004). A cytosolic glucosyltransferase is required for conversion of starch to sucrose in Arabidopsis leaves at night. Plant J..

[49] Niittyla T (2004). A previously unknown maltose transporter essential for starch degradation in leaves. Science.

[50] Moore B (2003). Role of the Arabidopsis glucose sensor HXK1 in nutrient, light, and hormonal signaling. Science.

[51] Rolland F, Baena-Gonzalez E, Sheen J (2006). Sugar sensing and signaling in plants: conserved and novel mechanisms. Annu. Rev. Plant Biol..

[52] Ruan YL (2014). Sucrose metabolism: gateway to diverse carbon use and sugar signaling. Annu. Rev. Plant Biol..

[53] Kötting O (2005). Identification of a novel enzyme required for starch metabolism in Arabidopsis leaves. The phosphoglucan, water dikinase. Plant Physiol..

[54] Tatge H, Marshall J, Martin C, Edwards EA, Smith AM (1999). Evidence that amylose synthesis occurs within the matrix of the starch granule in potato tubers. Plant Cell Environ..

[55] Uchiyama T, Watanabe K (2006). Improved inverse PCR scheme for metagenome walking. Biotechniques.

[56] Thompson JD, Gibson TJ, Plewniak F, Jeanmougin F, Higgins DG (1997). The CLUSTAL_X windows interface: flexible strategies for multiple sequence alignment aided by quality analysis tools. Nucleic Acids Res..

[57] Tamura K (2011). MEGA5: Molecular Evolutionary Genetics Analysis using maximum likelihood, evolutionary distance, and maximum parsimony methods. Mol. Biol. Evol..

[58] Vanderschuren H, Alder A, Zhang P, Gruissem W (2009). Dose-dependent RNAi-mediated geminivirus resistance in the tropical root crop cassava. Plant Mol. Biol..

[59] Zhang P, Potrykus I, Puonti-Kaerlas J (2000). Efficient production of transgenic cassava using negative and positive selection. Transgenic Res..

[60] Xu J, Duan XG, Yang J, Beeching JR, Zhang P (2013). Enhanced reactive oxygen species scavenging by overproduction of superoxide dismutase and catalase delays post-harvest physiological deterioration of cassava storage roots. Plant Physiol..

[61] Ritte G, Lorberth R, Steup M (2000). Reversible binding of the starch-related R1 protein to the surface of transitory starch granules. Plant J..

[62] Bradford MM (1976). A rapid and sensitive method for the quantitation of microgram quantities of protein utilizing the principle of protein-dye binding. Anal. Biochem..

[63] Knutson CA, Grove MJ (1994). Rapid method for estimation of amylose in maize starches. Cereal Chem..

