# Supplementary material for: Alpha-Glucan, Water Dikinase 1 Affects Starch Metabolism and Storage Root Growth in Cassava (Manihot esculenta Crantz)
Source: Sci Rep. 2017 Aug 29;7:9863. doi: 10.1038/s41598-017-10594-6 (PMC5575247; doi:10.1038/s41598-017-10594-6)
Supplement: Supplementary file 1 — Supplementary Information [file 41598_2017_10594_MOESM1_ESM.pdf]

Alpha-Glucan, Water Dikinase 1 Affects Starch Metabolism and Storage Root Growth in Cassava (*Manihot esculenta* Crantz). *Wenzhi Zhou, Shutao He, Maliwan Naconsie, Qiuxiang Ma, Samuel C. Zeeman, Wilhelm Gruissem and Peng Zhang*

SUPPLEMENTARY INFORMATION

Table S1 Primers used for T-DNA insertion and gene expression analyses.

Gene	Primer name and sequence
MeActin	AcFP: 5’-TGATGAGTCTGGTCCATCCA-3’
	AcRP: 5’-CCTCCTACGACCCAATCTCA-3’
MeGWD1	G1FP: 5’-ACCTCTGCATGGCTGTCCTGGTT-3’
	G1RP: 5’-GCACGGCCGGGATAGGCTCC-3’
MeGWD2	G2FP: 5’-CATGGCTGTGCTGATTCAAG-3’
	G2RP: 5’-GAAGCTCATTGCTCGTCCTG-3’
MeGWD3	G3FP: 5’-GCCGTCGAGCTGCTGGTGTGT-3’
	G3RP: 5’-CGCCAAGGAGTGCCCCGAGT-3’
MeGWD1	SRD5'FP: 5’-CACAAAGCAAGATACACGCTC-3’
	SRD5'RF: 5’-ACAAGCTGTGCTAAGTCCCT-3’

Table S2 Sequence feature of cassava GWDs.

Gene Name	CDS (bp)	Protein (aa)	Gene (bp)	Exon No.	Promoter (kb)	Source
MeGWD1	4230	1410	11120	33	>2	RIKEN
MeGWD2	3687	1229	18387	28	>2	AM560 genome & NCBI EST
MeGWD3	3585	1195	17430	18	>2	AM560 genome & RT-PCR

Table S3 Scales of cassava storage roots in different development stages.

Classification	Diameter (cm)
F (fibrous roots)	0.15~0.40 cm
D (developmental roots)	0.50~1.10 cm
M1 (mature storage roots 1)	1.80~2.10 cm
M2 (mature storage roots 2)	2.10~3.40 cm
M3 (mature storage roots 3)	3.40~4.20 cm

Table S4 Statistics analysis of root diameters in the wild type (WT) and G1i transgenic cassava.

Parameters		WT	G1i-12	G1i-17	G1i-28	G1i-31
Mean (cm)		2.9	2.3	2.1	2.4	2.4
Minimum (cm)		1.7	0.9	1.0	1.2	1.3
Maximum (cm)		4.3	4.0	3.4	3.5	3.9
quartiles (cm)	Lower quartile	2.2	1.8	1.5	2.1	1.9
	Median	2.8	2.3	2.0	2.5	2.5
	Upper quartile	3.5	2.8	2.6	2.8	2.9

Notes: Roots with diameter of lower quartile, median and upper quartile were used for analyzing sugar and starch content.

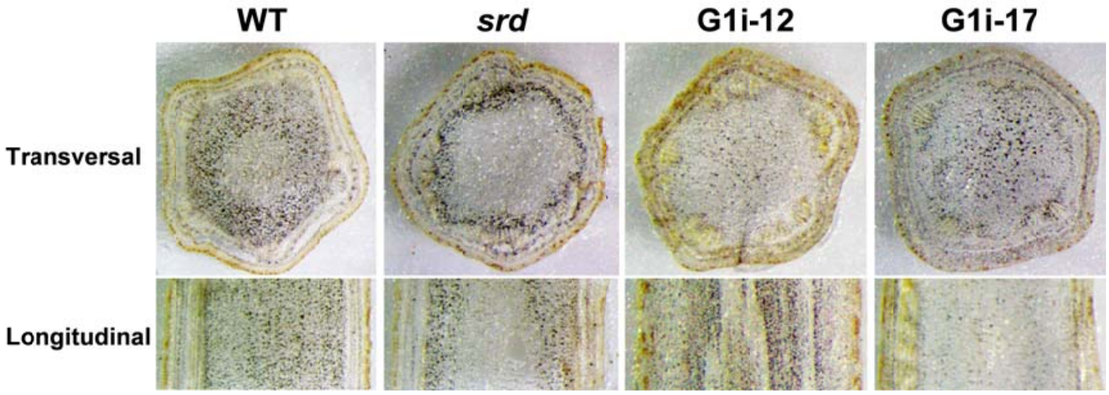

Figure S1 | Starch-iodine staining patterns in young stem sections of the wild type (WT), *srd* mutant and G1i transgenic cassava plants.

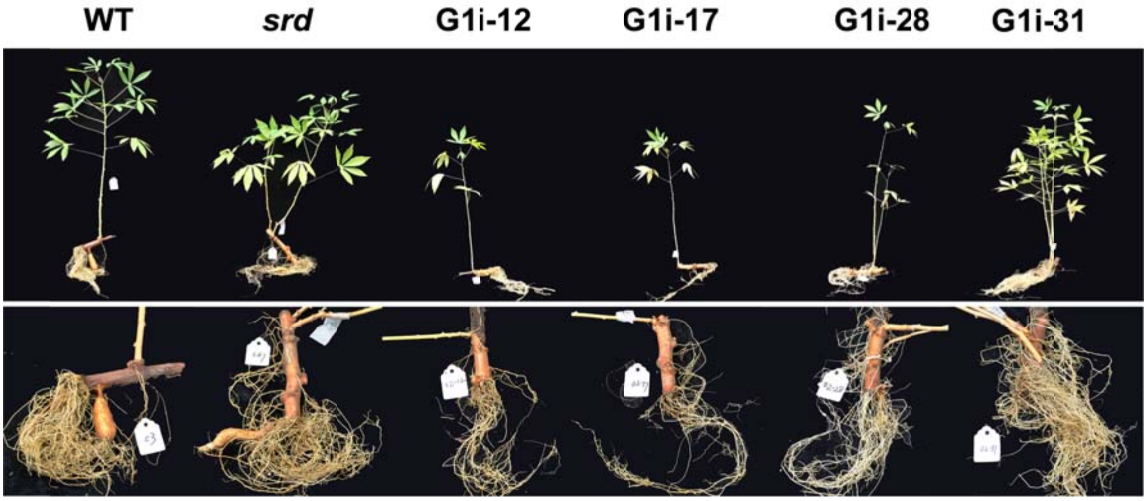

Figure S2 | Phenotype analysis of pot-grown cassava plants from mature stems after 3 months. The wild type (WT), *srd* mutant and four *MeGWD1* RNAi transgenic lines were used.

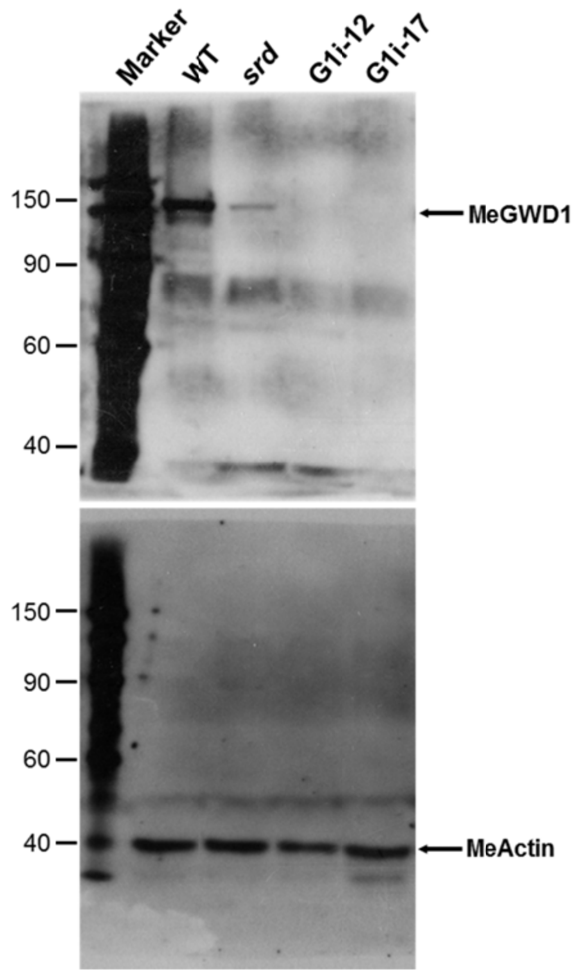

Figure S3 | Full image of Western blotting using antibody directed against MeGWD1 in the wild type (WT), *srd*, and two Gli transgenic cassava plants. The MeActin was used as a loading control.

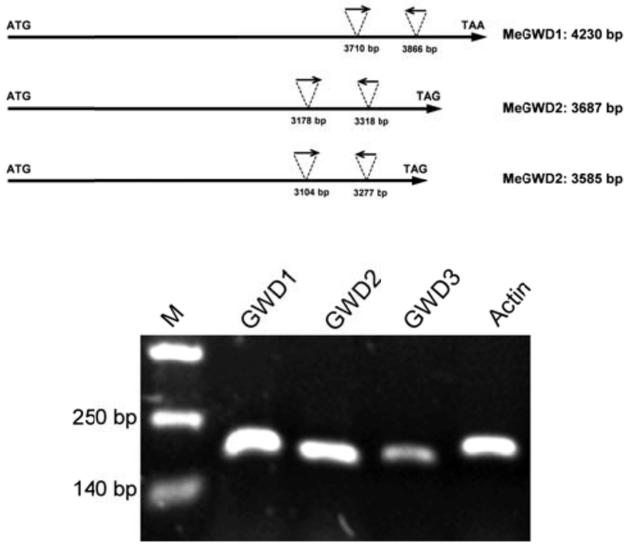

Figure S4｜Prime pairs used in the qRT-PCR reaction for gene expression of cassava GWD genes and their specificity confirmed by semi-quantitative RT-PCR assay.

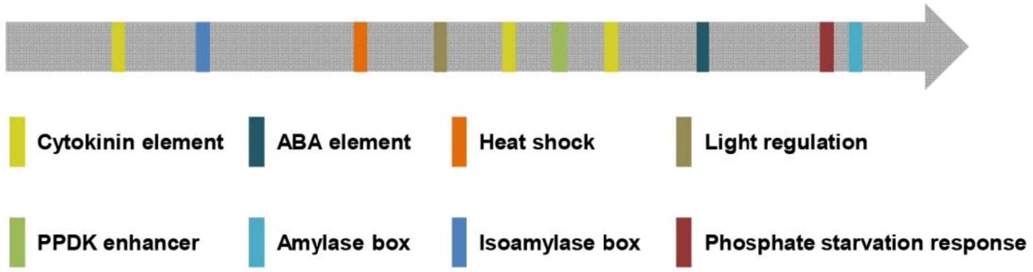

Figure S5｜The structure of the *MeGWD1* promoter.

The entire 2415 bp intergenic region upstream of the *MeGWD1* gene is displayed. A subset of the promoter elements identified with the PLACE tool (Higo et al. 1999) is indicated by color bars. The subset was selected to illustrate the diversity of signals potentially affecting GWD transcription.

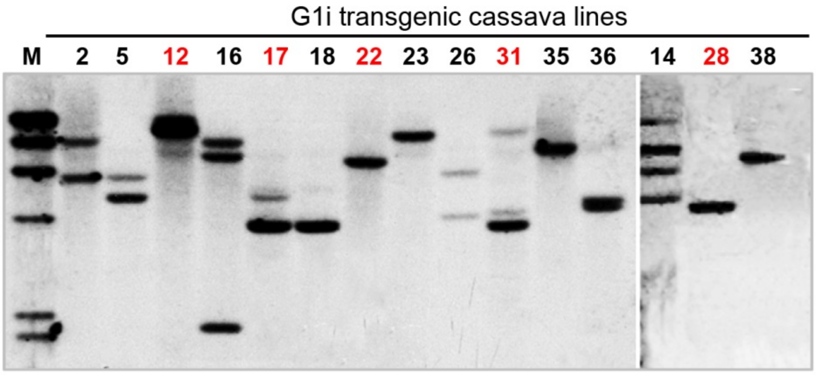

Figure S6︱Integration patterns of the T-DNA in the Gli transgenic cassava plants by Southern blot analysis.

Genomic DNA was digested with *Hin*dIII and hybridized with the digoxigenin (DIG)-labeled hygromycin phosphotransferase gene (*HPT*) probe. Red numbers were selected lines for following investigation.

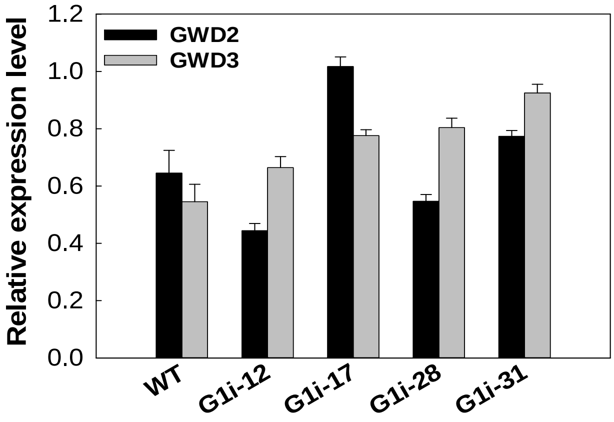

Figure S7︱Expression level of cassava *GWD2* and *GWD3* in wild type (WT) and G1i transgenic plant lines by qRT-PCR analysis.

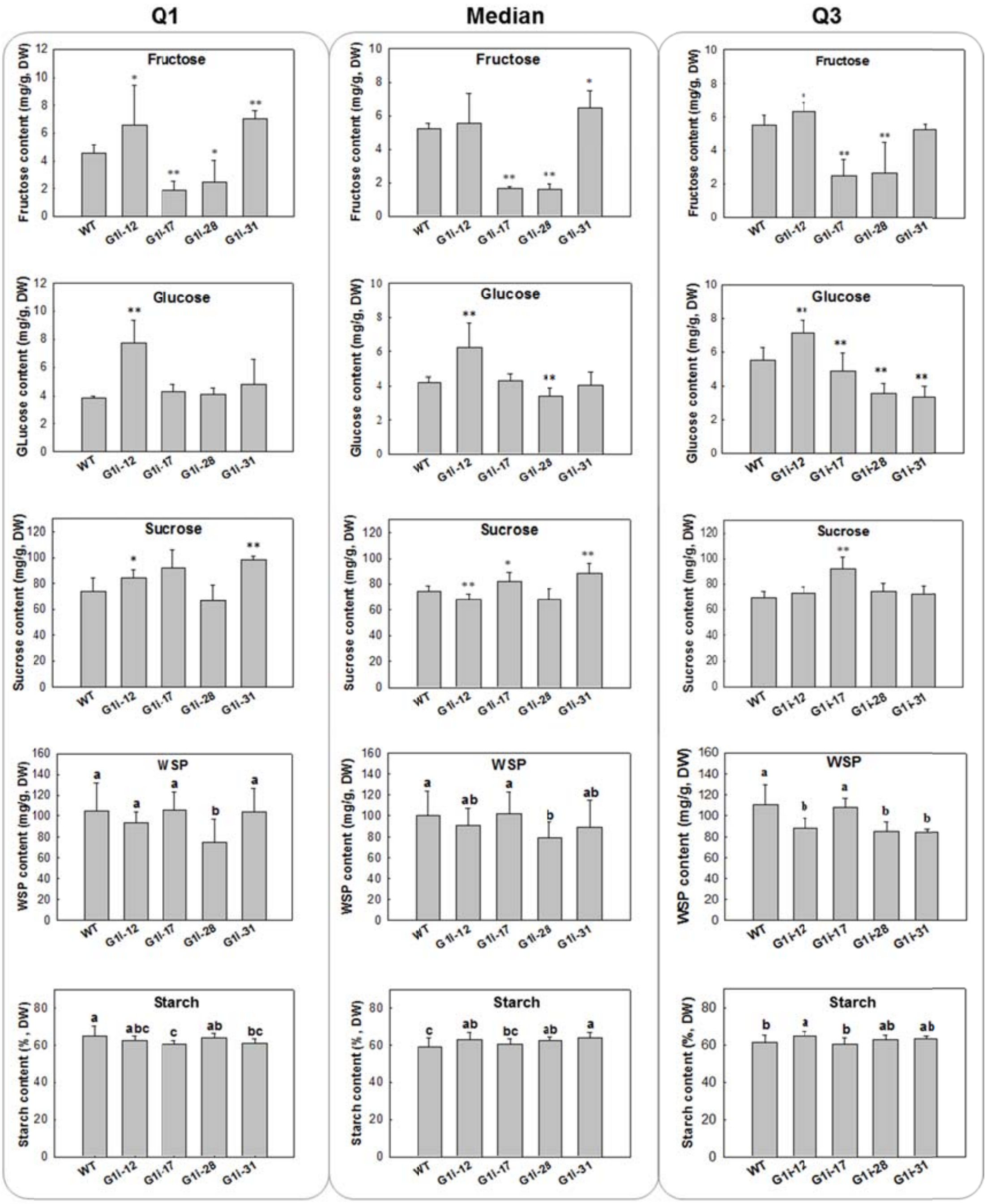

Figure S8｜**Sugar and starch contents of storage roots from the wild type (WT) and G1i transgenic cassava harvested from the field.**

Q1, roots with diameter of lower quartile according to roots diameter boxplot; Median, roots with diameter of lower quartile; Q3, roots with diameter of upper quartile. Data was presented as mean ± SD.

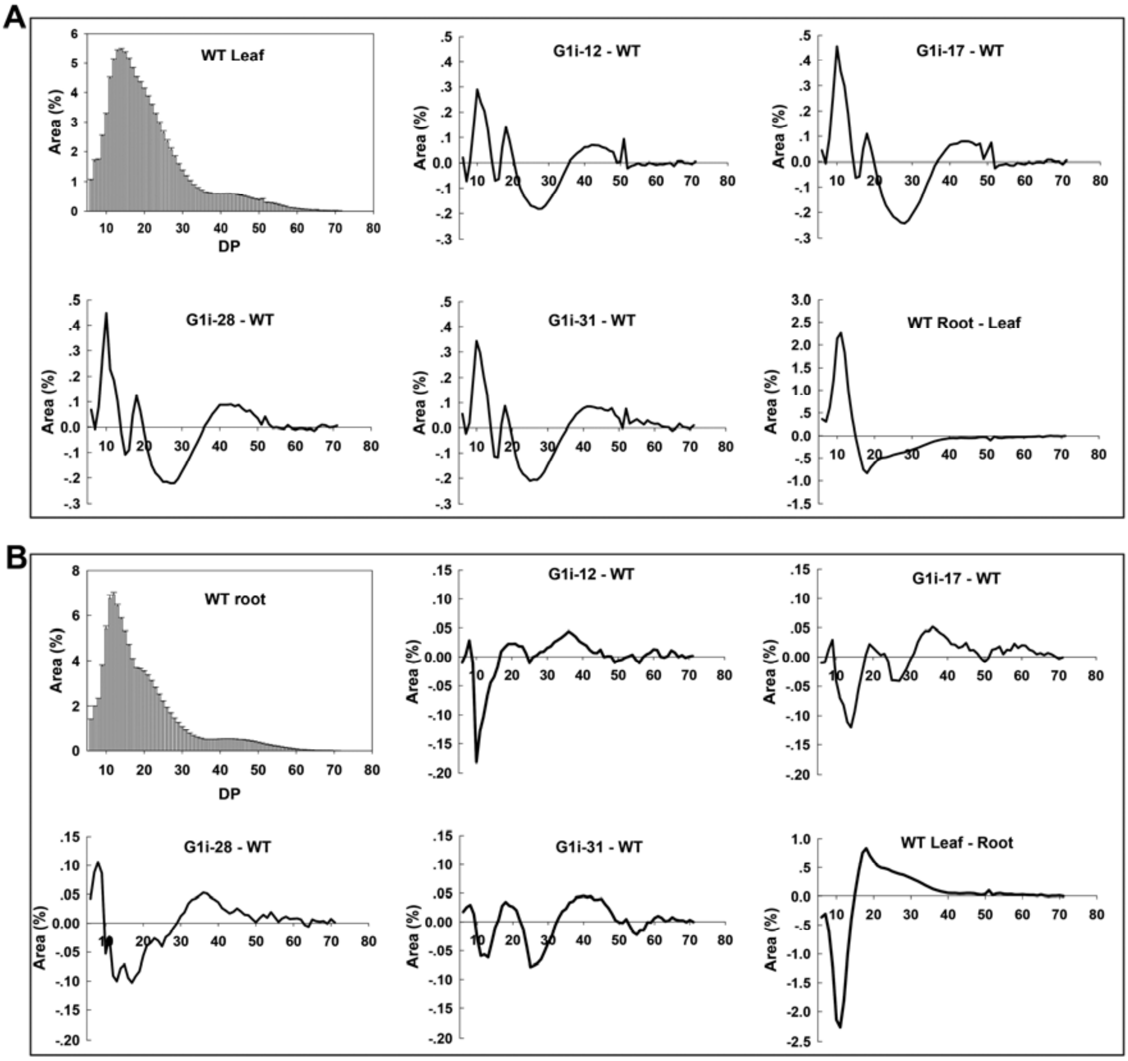

Figure S9｜Distributions of chain lengths of transient and storage starches.

(**A**) Differential profiles of transient starch. The frequency of chain length of transient starch from the wild type (WT) is normalized to total peak area. The normalized value for each chain length of transient starch of the G1i transgenic cassava or the WT storage starch is calculated by subtraction of the corresponding WT's value.

(**B**) Differential profiles of storage starch. The frequency of chain length of the storage starch from wild type (WT) is normalized to total peak area. The normalized value for each chain length of storage starch of the G1i transgenic cassava or the WT leaf starch is calculated by subtraction of the corresponding WT's value.
